# Multi-functional minor pilins coordinate type IV pilus assembly, adherence, motility, and DNA uptake in the pediatric pathogen *Kingella kingae*

**DOI:** 10.1128/mbio.02790-25

**Published:** 2025-10-13

**Authors:** Taylor A. Yount, Eric A. Porsch, Joseph W. St. Geme

**Affiliations:** 1 Children’s Hospital of Philadelphia 24178, Philadelphia, Pennsylvania, USA; 2 Perelman School of Medicine, University of Pennsylvania, Philadelphia, Pennsylvania, USA; University of California, Berkeley, Berkeley, California, USA

**Keywords:** type IV pili, minor pilins, adherence, twitching motility, natural competence

## Abstract

Type IV pili (T4P) are dynamic surface appendages that mediate adherence, motility, and DNA uptake in *Kingella kingae*, an important pediatric pathogen that causes osteoarticular infections, bacteremia, and endocarditis. While the major pilin subunit in *K. kingae* T4P is well characterized, the contribution of minor pilins to T4P structure and function remains unknown. Here, we used proteomics, molecular genetics, biochemical analyses, and structural modeling to identify and characterize all eight minor pilins in *K. kingae*. We identified a conserved operon of core minor pilin genes encoding FimT, PilV, PilW, PilX, and PilE that promotes surface piliation, adherence to epithelial cells, twitching motility, and natural transformation. Deletion of the *fimTpilVWXE* locus phenocopied loss of the PilC1 and PilC2 adhesins, and AlphaFold modeling combined with bacterial two-hybrid analysis suggested that FimT, PilV, PilW, and PilX form a complex at the pilus tip. The PilA2, ComP, and KK03_01180 minor pilins were dispensable for adherence and motility but promoted natural transformation and formed protein-protein interactions with the major pilin, suggesting that these proteins are incorporated throughout the pilus shaft. These findings support a new model for the architecture of the *K. kingae* type IV pilus, with distinct minor pilins localizing to different sites on the pilus fiber and mediating specialized functions essential for virulence.

**IMPORTANCE:**

*Kingella kingae* is an emerging pediatric pathogen and a leading cause of osteoarticular infections in children 6 months to 4 years of age. To establish infection, *K. kingae* relies on T4P, dynamic surface structures that mediate host cell adherence, motility, and DNA uptake. T4P are expressed by a wide range of bacterial pathogens beyond *K. kingae*, including *Pseudomonas aeruginosa*, *Neisseria gonorrhoeae*, *Neisseria meningitidis*, and *Legionella pneumophila*, among others. The type IV pilus is composed of pilin subunits, including a major pilin that displays significant antigenic diversity and low-abundance minor pilins that are highly conserved. This study demonstrates the importance of eight minor pilins in *K. kingae* virulence properties. Given the conservation of minor pilins across diverse bacterial species, targeting minor pilin complexes may provide a foundation for a new class of broad-spectrum antivirulence therapies that prevent bacterial colonization and disease.

## INTRODUCTION

Recent advances in molecular diagnostic techniques have established that the fastidious gram-negative bacterium *Kingella kingae* is the primary causative agent of septic arthritis and osteomyelitis in young children in many countries (1). In addition, *K. kingae* is an important cause of potentially life-threatening endocarditis in children and adults (2) (3). *K. kingae* initiates infection by colonizing the oropharynx, and approximately 70% of children are colonized at some point during the first 3 years of life (4). Following the breach of the oropharyngeal epithelium, likely mediated by a potent RTX toxin and/or a viral co-infection, *K. kingae* enters the bloodstream and disseminates to distant body sites (5–8). Most invasive *K. kingae* infections are treated effectively with β-lactam antibiotics, although β-lactamase production has recently been described in *K. kingae* clinical isolates (9, 10). There are currently no strategies for the prevention of *K. kingae* invasive disease.

To successfully colonize the oropharynx, *K. kingae* produces hair-like surface fibers called type IV pili (T4P) (11). *K. kingae* expresses a class of T4P called type IVa pili (T4aP), which extend and retract to carry out high levels of adherence to epithelial cells, a form of surface movement called twitching motility, and natural competence (12, 13). T4P are expressed by many bacterial pathogens, and their biogenesis machinery is highly conserved (14, 15). In *K. kingae*, an operon homologous to the *Pseudomonas aeruginosa pilMNOPQ* cluster is predicted to encode components of the pilus machinery. The PilMNOP alignment complex is anchored to the inner membrane and spans the periplasm, where the PilQ secretin ring is embedded in the outer membrane (16). At the inner membrane, small protein subunits called major pilins are polymerized and depolymerized to rapidly push and pull the fiber through the PilQ pore (17). Pilus activity is driven by cytoplasmic ATPase motors called PilF and PilT, which promote pilus extension and pilus retraction, respectively (13, 18, 19). Across T4P-expressing bacteria, major pilins have a characteristic “lollipop” structure with a hydrophobic N-terminal alpha helix that inserts into the core of the pilus and a C-terminal globular head domain that is externally exposed (20). The *K. kingae* major pilin, known as PilA1, displays significant antigenic diversity across clinical isolates, particularly within the surface-exposed C-terminus (21). Attached to the pilus fiber are two large adhesins called PilC1 and PilC2, which have homologs in other bacterial species, including PilY1 in *P. aeruginosa*, PilY1 in *Legionella pneumophila*, and PilC1 and PilC2 in *Neisseria meningitidis* and *Neisseria gonorrhoeae* (constituting the PilY/PilC family) (22–26). *K. kingae* PilC1 and PilC2 directly mediate adherence to epithelial cells and are important for twitching motility and natural transformation (27).

Beyond the major pilin subunit, T4P-expressing bacteria produce several low-abundance pilins known as minor pilins (28). These proteins share the same characteristic lollipop structure as the major pilin but are much less abundant in the pilus fiber (28). The minor pilins are a versatile class of proteins that promote pilus formation, DNA binding, aggregation, twitching motility, and adherence in other systems (28). In *P. aeruginosa* and *Myxococcus xanthus*, a set of conserved minor pilins called core minor pilins is required to prime pilus assembly and is predicted to form a complex at the tip of the extended pilus (29, 30). The PilV minor pilin in *N. meningitidis* is incorporated throughout the pilus and directly adheres to human epithelial cells (31). The ComP minor pilin in the pathogenic *Neisseria* species facilitates natural competence by directly binding and taking up exogenous DNA (32–38). Though often overlooked, minor pilins can be critical for pilus function and may be good candidates as therapeutic targets, given that they are surface exposed and often highly conserved across invasive clinical isolates. Although several T4P crystal structures have been reported, no crystal structure of a complete pilus that identifies specific minor pilin subunits has been generated (16, 28, 39, 40), representing a significant gap in our understanding of how and where minor pilins are incorporated into T4P. In *K. kingae*, earlier work established that PilC1 and PilC2 are crucial pilus-associated adhesins (27). However, it is largely unknown how other pilus-associated proteins such as minor pilins contribute to pilus structure and function in this species.

In this study, we set out to identify minor pilins in *K. kingae* and characterize their contribution to T4P formation, adherence, twitching motility, and natural competence. We employed proteomic analysis of purified pili, phenotypic analyses of minor pilin mutants, and biochemical analyses to gain a comprehensive understanding of the roles of *K. kingae* minor pilins in T4P structure and function.

## RESULTS

### The FimT, PilV, PilW, PilX, and PilE minor pilins promote pilus biogenesis

**Fig 1:**
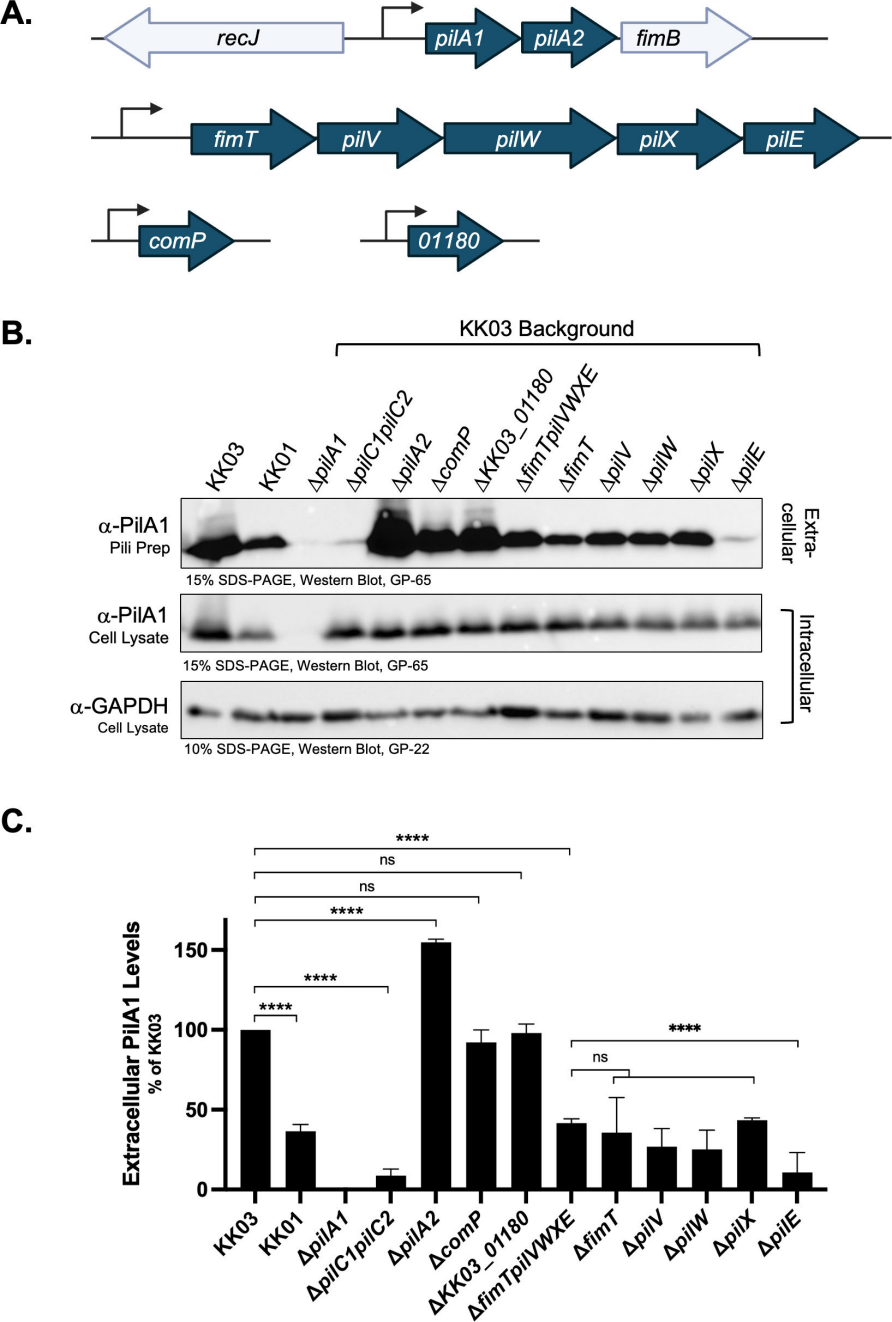
(**A**) Schematic of genes encoding pilin-like proteins (blue) in the KK03 genome. The *pilA1* and *pilA2* genes are co-transcribed under the control of a single promoter. The *fimTpilVWXE* locus encodes five minor pilins of distinct sizes under the control of a single promoter. The *comP* and *KK03_01180* genes are located at separate locations in the genome, far from other T4P genes. (**B**) Purified pili and whole cell lysates from *K. kingae* strains were boiled and separated by SDS-PAGE to assess for extracellular pili production and intracellular PilA1 expression; pili preps and cell lysates were probed with PilA1 antiserum (GP-65). As a loading control, cell lysates were probed with GAPDH antiserum (GP-22). (**C**) Piliation levels were quantitated using densitometry to measure the PilA1 band intensity in the Western blots on pilus preparations (*n* = 3). The band intensity is reported as a percentage of wild type (KK03). Error bars represent standard errors of the mean (*n* = 3). **** indicates significance of *P* < 0.0001 as determined by one-way analysis of variance with a Dunnett correction for multiple comparisons. ns, no statistical difference.

In order to identify the proteins present in the type IV pilus, mass spectrometry was performed on purified pili from the wild-type (WT) *K. kingae* strain KK03, a stable derivative of septic arthritis isolate 269-492 that produces high levels of T4P. T4P were purified by shearing the pili from the bacterial surface with a handheld tissue homogenizer and subjecting the pili to ultracentrifugation and ammonium sulfate precipitation to remove contaminants. As expected, the 16 kDa major pilin PilA1 was detected at high levels in the purified pili (Table S1) (Fig. S1). In addition, PilA2, PilC1, PilC2, FimT, PilV, PilW, PilX, PilE, PilT, PilU1, PilU2, PilM, PilO, and PilQ were all identified in the purified pili (Table S1). Of these, PilA1, PilA2, FimT, PilW, PilV, PilX, and PilE were predicted by AlphaFold 3 to form a pilin-like structure (Fig. S2). Using the bioinformatics tool TXSScan and AlphaFold 3 structural prediction (41, 42), we identified two additional predicted pilins: ComP and KK03_01180 (Table S1, Fig. S2). Examination of the KK03 genome revealed that the genes encoding FimT, PilV, PilW, PilX, and PilE (*fimT*, *pilV*, *pilW*, *pilX*, and *pilE*) are clustered together under a single promoter; the gene encoding *pilA2* is in the same operon as *pilA1* (11); and the genes encoding ComP and KK03_01180 (*comP* and *KK03_01180*) are located far from other pili-related genes in the genome (Fig. 1A) and appear to have their own promoters. The FimT, PilV, PilW, PilX, and PilE proteins show some sequence similarity to the gene products of the loci encoding core minor pilins in *P. aeruginosa*, *M. xanthus*, and pathogenic *Neisseria* species, especially within the N-terminal regions of the pilins (Fig. S3). Furthermore, BLAST searches across the publicly available *K. kingae* genome database revealed that the locus encoding the FimT, PilV, PilW, PilX, and PilE predicted pilins in KK03 is highly conserved across clinical isolates of *K. kingae* (~98% to 100% identity), suggesting that these proteins may be critical for T4P structure and/or function. Of the predicted *K. kingae* pilins, PilA2 displayed the highest levels of sequence identity and similarity to the PilA1 major pilin (Table S1), sharing 50% identity and 65% similarity. Among the eight predicted minor pilins, structural predictions via AlphaFold 3 combined with pairwise structure alignments revealed that PilA2, KK03_01180, ComP, and PilE are the most similar in size and structure compared to PilA1 (Fig. S4). FimT, PilV, PilW, and PilX have high structural similarity with PilA1 within the N-terminal alpha helix but differ from PilA1 in size and predicted conformation in their C-termini (Fig. S4).

Previous work has demonstrated that PilA2 is dispensable for pilus biogenesis (11). To understand the role of *KK03_01180* and *comP* in surface piliation, deletion mutants were created and surface pili were sheared and purified from each mutant. For strains Δ*KK03_01180* and Δ*comP*, high levels of PilA1 were detected within the purified pili samples (Fig. 1B and C), suggesting that they are dispensable for pilus biogenesis. To examine the roles of the FimT, PilV, PilW, PilX, and PilE predicted pilins in T4P formation, we generated a *K. kingae* strain with a full deletion of the *fimTpilVWXE* locus (Δ*fimTpilVWXE*). Given the requirement of core minor pilins for priming pilus biogenesis in other T4P-expressing species (29, 30), we predicted that the Δ*fimTpilVWXE* mutant would not produce T4P. However, as shown in Fig. 1B and C, strain Δ*fimTpilVWXE* exhibited significant levels of surface piliation, albeit at slightly lower levels compared to the wild-type parent strain KK03. Importantly, there was no difference in PilA1 levels within the cell lysates of KK03 and Δ*fimTpilVWXE*, suggesting that the decrease in Δ*fimTpilVWXE* surface piliation is due to a defect in pilus biogenesis, not a change in the amount of PilA1 produced (Fig. 1B). Insertional inactivation of the *pilT* gene (Δ*fimTpilVWXEpilT*), which encodes an intracellular retraction ATPase, resulted in significantly increased levels of pili compared to Δ*fimTpilVWXE* (Fig. S5). This result suggests that the FimT, PilV, PilW, PilX, and PilE minor pilins may promote high levels of surface piliation by counteracting retraction of the pilus, a function that is associated with the PilY/PilC family adhesins (16, 27, 43). Chromosomal complementation of the Δ*fimTpilVWXE* deletion resulted in a modest increase in surface piliation levels compared to the mutant, further supporting the conclusion that the FimT, PilV, PilW, PilX, and PilE minor pilins promote high levels of surface piliation (Fig. S6). Examination of the Δ*fimTpilVWXE* mutant by negative-staining transmission electron microscopy (TEM) revealed the presence of T4P with similar morphology to pili in KK03, suggesting that the FimT, PilV, PilW, PilX, and PilE core minor pilins are not required to prime pilus biogenesis in *K. kingae* (Fig. S7). Additionally, the levels of pili observed in the TEM micrographs were decreased for both the Δ*fimTpilVWXE* and Δ*pilC1pilC2* strains compared to KK03, consistent with the results of the pili purification in Fig. 1B; Fig. S7.

To assess the necessity of individual pilins encoded by the *fimTpilVWXE* locus for pilus biogenesis and surface piliation, we generated five separate in-frame deletion mutants with each of the genes in the locus replaced with the *ermC* erythromycin resistance gene (Δ*fimT*, Δ*pilV*, Δ*pilW*, Δ*pilX*, and Δ*pilE*). Each of the minor pilin deletion mutants displayed a defect in surface piliation, albeit to different levels (Fig. 1B and C). The Δ*pilE* mutant displayed significantly lower levels of surface piliation compared to the other four core minor pilin mutants, suggesting that PilE may have a distinct and more significant role in promoting pilus biogenesis compared to FimT, PilV, PilW, and PilX (Fig. 1B and C). Chromosomal complementation of each individual core minor pilin under control of its native promoter resulted in an increase in surface piliation levels produced by the Δ*fimT*, Δ*pilW*, Δ*pilX*, and Δ*pilE* deletion mutants (Fig. S6). Chromosomal complementation of the Δ*pilV* deletion did not have a noticeable impact on surface piliation levels (Fig. S6). Overall, these results indicate that all of the FimT, PilV, PilW, PilX, and PilE core minor pilins promote high levels of surface piliation but are not individually required for pilus biogenesis to occur.

### Each of the FimT, PilV, PilW, PilX, and PilE minor pilins is essential for adherence and twitching motility

**Fig 2:**
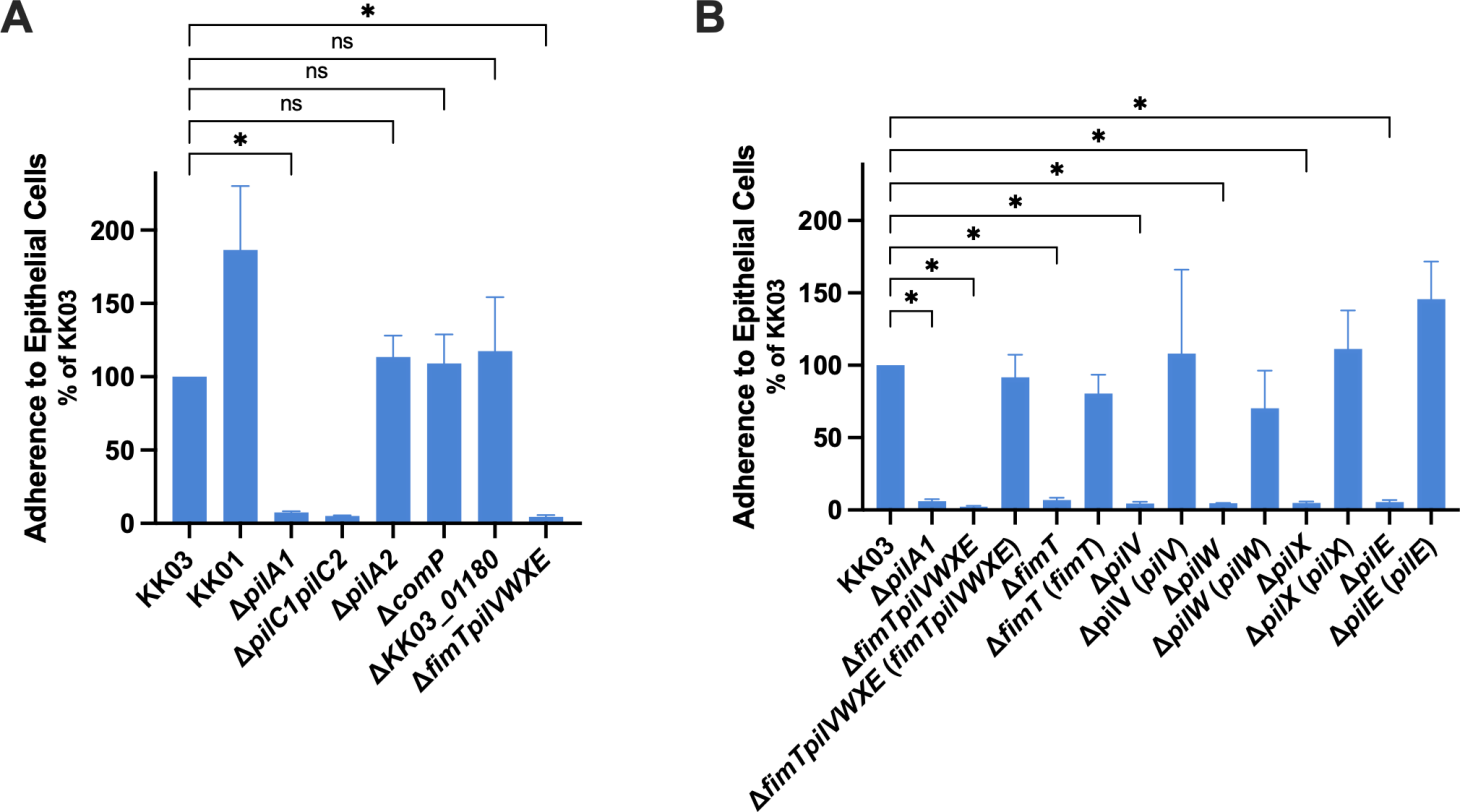
Quantitative adherence assay with minor pilin mutants (**A**) and core minor pilin complement strains (**B**). *K. kingae* strains were added to monolayers of Chang epithelial cells of HeLa origin and evaluated for adherence using CFU plating. Adherence levels are reported as a percentage of wild type (KK03). Error bars represent standard errors of the mean (*n* = 3). * indicates significance of *P* < 0.05 as determined by one-way analysis of variance with a Dunnett correction for multiple comparisons. ns, no statistical difference.

An important function of *K. kingae* type IV pili is to mediate adherence to epithelial cells, a process that is dependent on the T4P-associated PilC1 and PilC2 adhesins. Previous work has demonstrated that PilA2 is dispensable for adherence to Chang epithelial cells (11). However, the role of the remaining *K. kingae* minor pilins in adherence to epithelial cells was previously uncharacterized. To investigate the adherence properties mediated by pili produced by strain Δ*fimTpilVWXE*, we performed adherence assays using epithelial monolayers. As shown in Fig. 2A, Δ*fimTpilVWXE* exhibited negligible levels of adherence, comparable to adherence by a non-piliated strain (Δ*pilA1*) and a strain lacking both PilC1 and PilC2 (Δ*pilC1pilC2*). To control for the influence of pilus density on adherence phenotypes, strain KK01 was included in this analysis, as KK01 is a stable colony variant of clinical isolate 269-492 that produces sparse but functional pili (44). Despite the lower level of pili (Fig. 1B), KK01 displays higher overall adherence compared to the hyper-piliated strain KK03 (Fig. 2A), suggesting that piliation level *per se* does not correlate with adherence in this model. Complementation of the Δ*fimTpilVWXE* deletion restored adherence to wild-type levels, further signifying that the FimT, PilV, PilW, PilX, and PilE minor pilins are required for *K. kingae* adherence to epithelial cells (Fig. 2B). Adherence levels were negligible for Δ*fimT*, Δ*pilV*, Δ*pilW*, Δ*pilX*, and Δ*pilE*, and adherence levels for the chromosomal complements of these mutants were wild type, suggesting that each of the FimT, PilV, PilW, PilX, and PilE pilins is required for adherence and may be dependent on the others to carry out this function (Fig. 2B). Quantification of adherence by the Δ*KK03_01180* and Δ*comP* mutants revealed wild-type levels of adherence, suggesting that the *KK03_01180* and *comP* products are not required for adherence (Fig. 2A).

**Fig 3:**
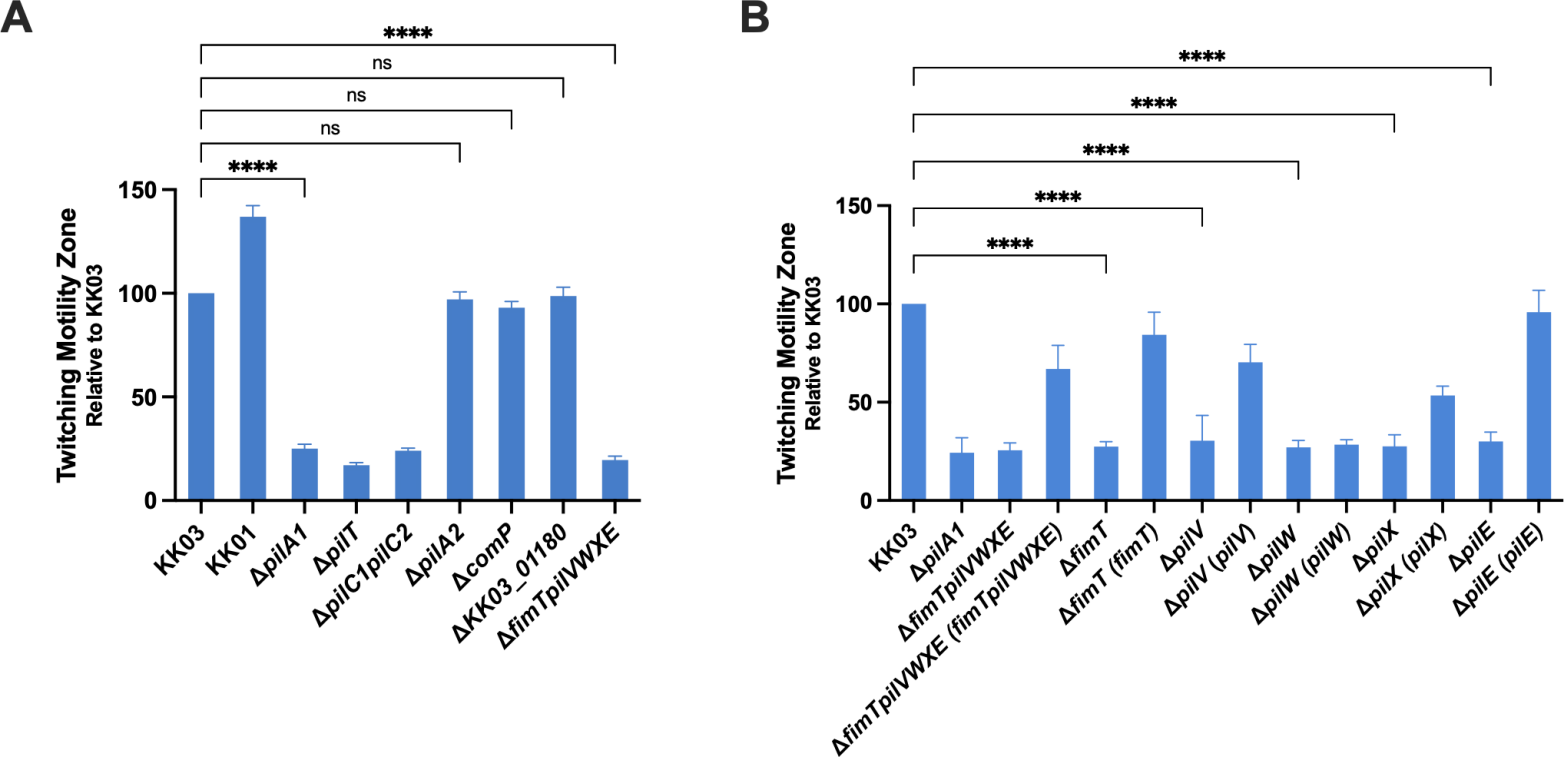
Twitching motility assays with minor pilin mutants (**A**) and core minor pilin complement strains (**B**). *K. kingae* strains were stab inoculated into the center of low-agar chocolate plates, and the zone of twitching was quantified by measuring the diameter of bacterial spread as visualized by crystal violet staining. The average diameter of the twitching zones is reported as a percentage of wild type (KK03). Error bars represent standard errors of the mean (*n* = 3). **** indicates significance of *P* < 0.0001, as determined by one-way analysis of variance with a Dunnett correction for multiple comparisons. ns, no statistical difference.

Twitching motility is a dynamic process, requiring pili to be able to extend, adhere, and retract to pull the bacteria along a surface (45). To assess whether the pili produced in Δ*fimTpilVWXE* possess this property, we performed twitching motility assays on low-agar chocolate plates. As shown in Fig. 3A, Δ*fimTpilVWXE* exhibited negligible twitching motility, similar to the levels exhibited by strains that are non-piliated (Δ*pilA1*), non-retractile (Δ*pilT*), or lacking both major adhesins (Δ*pilC1pilC2*). The high level of twitching motility exhibited by KK01 demonstrates that for WT strains, low levels of surface piliation are not correlated with decreased levels of twitching motility (Fig. 3A). Chromosomal complementation of the *fimTpilVWXE* locus restored twitching motility (Fig. 3B). Additionally, Δ*fimT*, Δ*pilV*, Δ*pilW*, Δ*pilX*, and Δ*pilE* exhibited negligible levels of twitching motility, suggesting that expression of each of the FimTPilVWXE pilins is required for twitching to occur (Fig. 3B). Chromosomal complementation of *fimT*, *pilV*, and *pilE* restored high levels of twitching motility. Interestingly, chromosomal complementation of *pilX* resulted in a low level of twitching, and complementation of *pilW* did not restore twitching motility at all. The levels of twitching motility in the Δ*pilA2*, Δ*KK03_01180*, and Δ*comP* mutants were not significantly different from KK03, suggesting that PilA2, KK03_01180, and ComP are dispensable for twitching motility (Fig. 3A).

### FimT, PilV, PilW, PilX, and ComP are required for natural transformation in *K. kingae*

**Fig 4:**
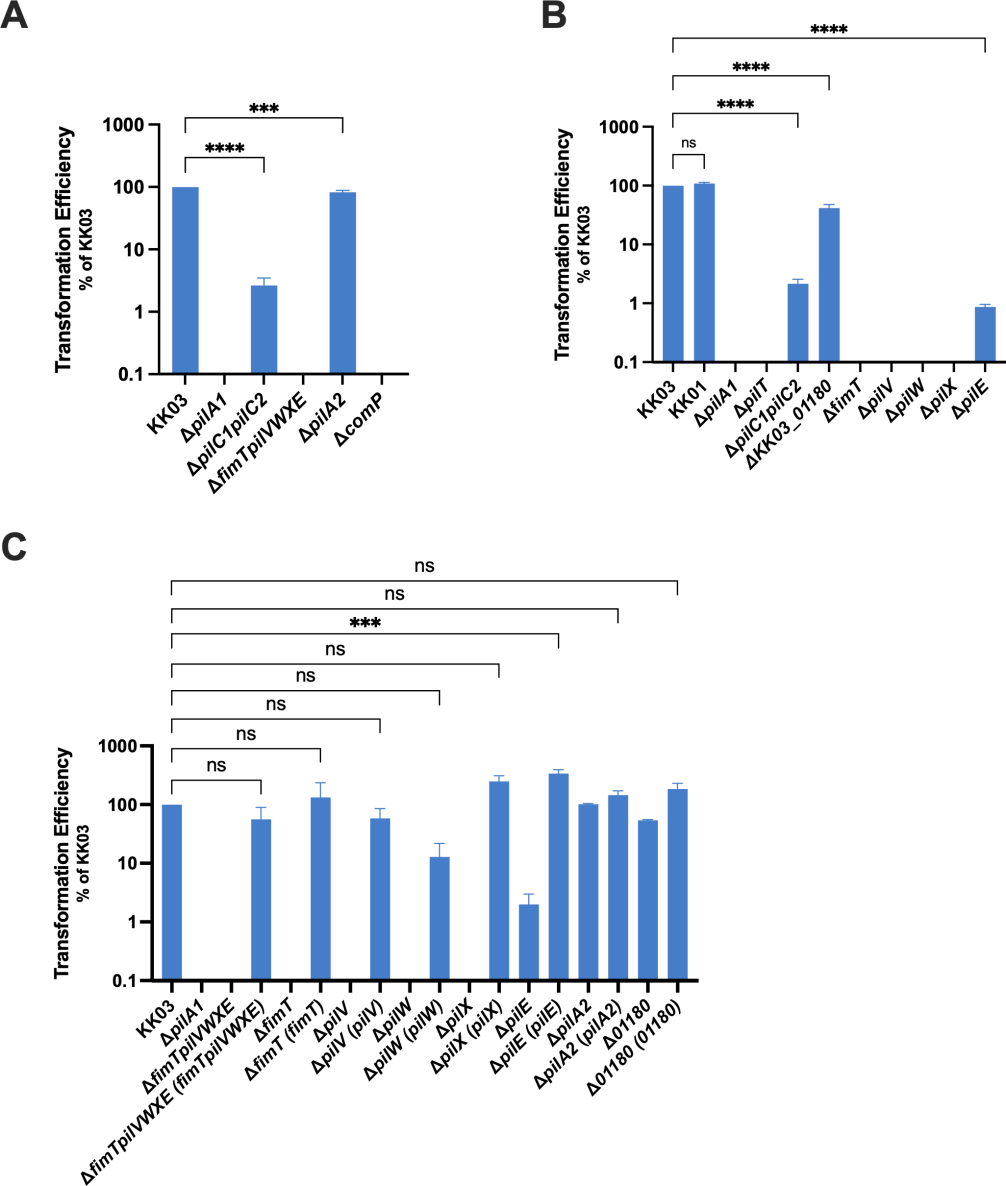
Quantitative natural transformation assays with minor pilin mutants (**A** and B) and minor pilin complement strains (**C**). *K. kingae* strains were naturally transformed with a plasmid containing an erythromycin (**A**), kanamycin (**B**), or tetracycline (**C**) resistance cassette designed to replace the *knh* gene in the chromosome. Transformation efficiency was calculated as a ratio of antibiotic-resistant CFU to inoculum CFU. The transformation efficiency is reported as a percentage of wild type (KK03). Error bars represent standard errors of the mean (*n* = 3.) *** indicates significance of *P* < 0.001 and **** indicates significance of *P* < 0.0001 as determined by one-way analysis of variance with a Dunnett correction for multiple comparisons. ns, no statistical difference.

In the process of natural competence, *K. kingae* T4P can readily take up exogenous DNA, which can be used nutritionally or can be incorporated into the chromosome via homologous recombination. As a proxy for natural competence, we measured the natural transformation efficiency of *K. kingae* strains by incubating them with plasmids encoding antibiotic resistance cassettes flanked by homologous *K. kingae* sequence to promote recombination and quantifying the number of successful transformants. Natural competence depends on the ability of a strain to extend pili, bind DNA, and retract pili to pull the DNA into the cell (46), and thus, non-piliated (Δ*pilA1*) and non-retractile (Δ*pilT*) strains are not naturally competent and are unable to undergo natural transformation (Fig. 4A and B). *K. kingae* natural transformation has been shown to be partially dependent on expression of PilC1 and PilC2 (27). Consistent with these findings, we observed that the transformation efficiency in the Δ*pilC1pilC2* mutant is only about 2% of the observed transformation efficiency for KK03 (Fig. 4A and B). The Δ*fimTpilVWXE* mutant was completely deficient in transformation, consistent with the observation that this strain is unable to twitch and potentially has altered pilus extension and retraction dynamics (Fig. 4A). Complementation of the Δ*fimTpilVWXE* deletion restored natural transformation (Fig. 4C). The findings with Δ*fimTpilVWXE* represent the first example where we observed a significant difference in phenotype between strains Δ*fimTpilVWXE* and Δ*pilC1pilC2*, suggesting that the FimT, PilV, PilW, PilX, and PilE pilins may have roles distinct from the PilC proteins in natural transformation. There were no transformants recovered from strains Δ*fimT*, Δ*pilV*, Δ*pilW*, and Δ*pilX* (Fig. 4B). Surprisingly, transformants were recovered from the Δ*pilE* mutant, and this strain exhibited a transformation efficiency level that was about 1% of KK03, similar to the level observed for the Δ*pilC1pilC2* strain (Fig. 4B). These results suggest that FimT, PilV, PilW, and PilX are required for natural transformation and that PilE promotes but is not essential for this process. Chromosomal complementation of Δ*fimT*, Δ*pilV*, Δ*pilW*, and Δ*pilX* restored transformation efficiency to wild-type levels (Fig. 4C). Interestingly, chromosomal complementation of the Δ*pilE* mutant resulted in a significant increase in transformation efficiency, reaching levels approximately threefold greater than observed for wild type (Fig. 4C). Altogether, these findings suggest that the core minor pilins promote natural transformation and that PilE may have a role in natural transformation that is distinct from FimT, PilV, PilW, and PilX. Based on the known role of ComP in *Neisseria* species, we hypothesized that the *K. kingae* ComP homolog would be required for natural transformation to occur. Consistent with this hypothesis, there were no transformants recovered from the Δ*comP* mutant in the natural transformation assay (Fig. 4B). Multiple attempts to generate a chromosomal complement of the Δ*comP* mutation were made but were ultimately unsuccessful. Δ*pilA2* and Δ*KK03_01180* exhibited transformation efficiencies that were 82% and 42% of those observed for KK03, respectively, suggesting that PilA2 and KK03_01180 have modest roles in promoting natural transformation (Fig. 4A and B). Chromosomal complementation of the Δ*pilA2* and Δ*KK03_01180* deletions restored transformation efficiencies to wild-type levels (Fig. 4C). Overall, these results demonstrate that all eight of the *K. kingae* minor pilins (PilA2, KK03_01180, ComP, FimT, PilV, PilW, PilX, and PilE) promote *K. kingae* natural transformation.

### Several minor pilins interact and may form complexes within the type IV pilus

**Fig 5:**
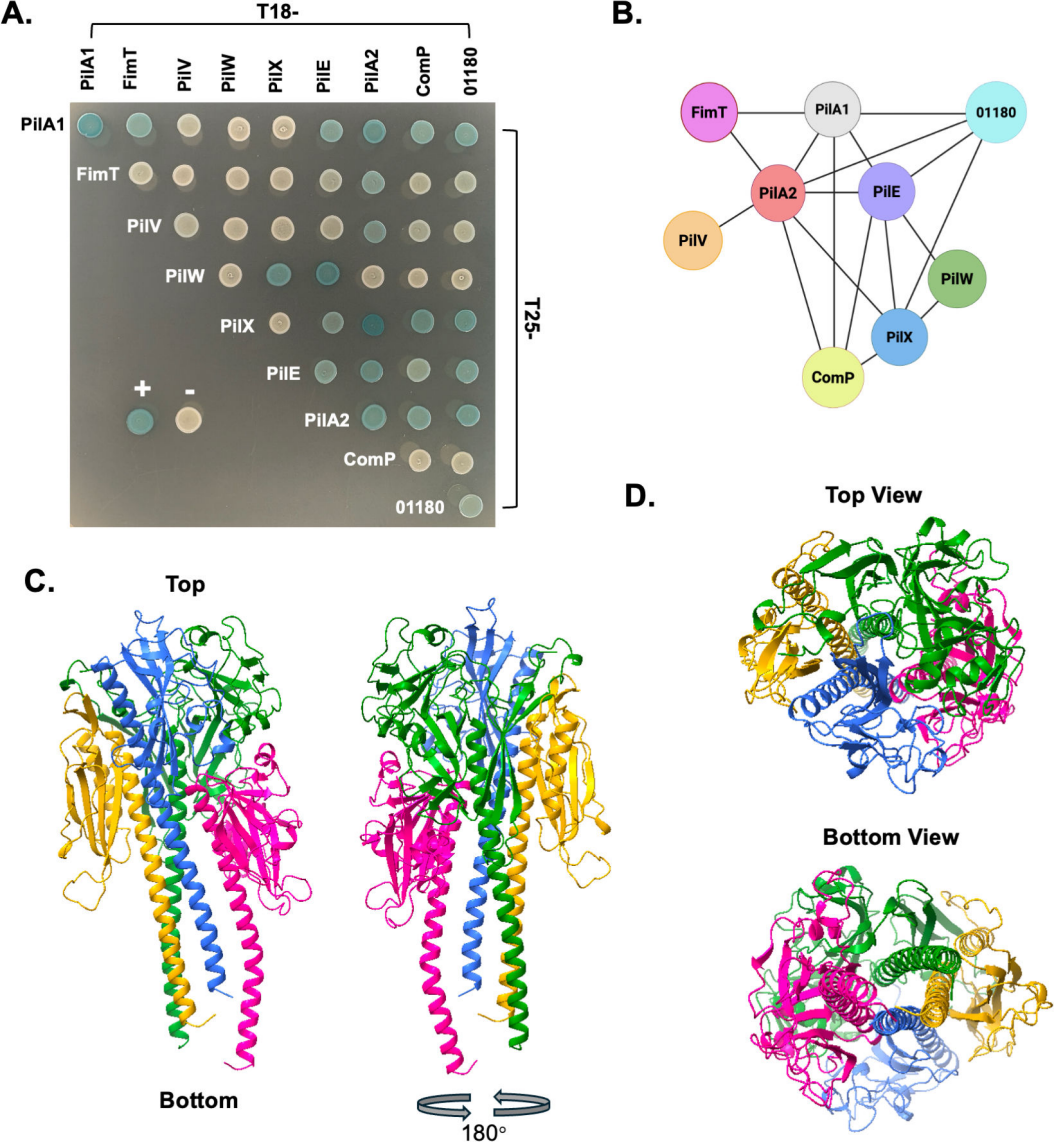
Analysis of protein-protein interactions between *K. kingae* pilins. (**A**) Bacterial two-hybrid spotting assay. The positive control (+) includes the T18 and T25 fragments of the CyaA adenylate cyclase fused to leucine zipper proteins, which are known to form a strong interaction (blue). The negative control (−) includes only the empty vectors encoding the T18 and T25 fragments, which do not interact (white). The pilins labeled in each row indicate that the bacteria in the spot were transformed with a plasmid encoding the T25 fragment fused to that pilin. The pilins labeled in each column indicate that the bacteria in the spot were transformed with a plasmid encoding the T18 fragment fused to that pilin. (**B**) Schematic demonstrating interactions between the pilins in the bacterial two-hybrid spotting assay. Self-interactions are not shown. (**C**) AlphaFold 3 Multimer-predicted structure of a pilin complex containing FimT (pink), PilV (orange), PilW (green), and PilX (blue). The structure on the left was rotated 180° (right) to properly display all proteins within the predicted structure. (**D**) Top and bottom views of the predicted structure shown in panel C.

The reliance on each pilin encoded by the *fimTpilVWXE* locus for adherence and twitching motility suggests that these minor pilins may assemble into a complex within T4P that is essential to carry out these functions. To test this possibility, we utilized a bacterial two-hybrid (BACTH) system to evaluate protein-protein interactions between T4P proteins. The T18 and T25 fragments of an adenylate cyclase were fused to the full-length mature major and minor pilins. Plasmids encoding these fusion proteins were then co-transformed into the reporter strain BTH101 and spotted onto luria-bertani (LB) agar containing 5-bromo-4-chloro-3-indolyl-β-D-galactopyranoside (X-gal) to observe a white to blue color change indicative of a protein-protein interaction (47). Consistent with our hypothesis of a core minor pilin complex, we observed interactions between PilW-PilX, PilW-PilE, and PilX-PilE (Fig. 5A and B). Interestingly, PilV, PilW, and PilX did not interact with PilA1, suggesting that they may exist in a complex within the pilus that is isolated from the major pilin. In contrast, FimT interacted with PilA1, suggesting that it may bridge interactions between the core minor pilin complex and the bulk of the pilus (Fig. 5A and B). Given the similarity in predicted molecular mass (15–17 kDa) and structure between PilA2, PilE, KK03_01180, ComP, and the major pilin (Fig. S4), we hypothesized that these minor pilins are incorporated throughout the pilus fiber. In support of this hypothesis, PilE, PilA2, ComP, and KK03_01180 interacted with PilA1 in the BACTH assay (Fig. 5A and B). Several additional interactions were observed between these similarly sized pilins, including PilA2-PilE, PilA2-ComP, and PilA2-01180 (Fig. 5A and B). Interestingly, we also observed interactions between PilA2, ComP, and 01180 and some of the larger core minor pilins—FimT, PilV, PilW, and PilX (22–38 kDa)—including FimT-PilA2, PilV-PilA2, PilX-PilA2, PilX-ComP, and PilX-01180, a surprising result, given the potential steric hindrance of the larger C-terminal globular domains when incorporated into the pilus polymer (Fig. 5A and B).

AlphaFold multimer was utilized to predict interactions between the eight minor pilins and PilA1 (48). The AlphaFold software was unable to fit PilA1, PilA2, ComP, 01180, and PilE into a complex that resembles the canonical structure of a pilus, which is characterized by interaction of the N-terminal hydrophobic alpha-helices in the center of the fiber with the C-terminal globular domains externally exposed. However, a high-confidence predicted structure was generated between *K. kingae* FimT, PilV, PilW, and PilX (Fig. 5C). This predicted structure places PilW, PilX, and PilV at the top of what may be a tip complex, with FimT binding below to the globular domain of PilW (Fig. 5C and D). This model is consistent with the interaction between FimT and PilA1 and the lack of interaction between PilW, PilX, and PilV with PilA1 in the BACTH assay, as the major pilin would likely polymerize below this tip complex, interacting with FimT (Fig. 5A and B).

## DISCUSSION

Type IV pili are dynamic surface appendages that extend and retract to mediate important bacterial behaviors, including host cell adherence, twitching motility, and DNA uptake (26, 27, 30, 49–53). In this study, we have examined the function of the minor pilin proteins in *K. kingae*, establishing that a set of core minor pilins—FimT, PilV, PilW, and PilX—form a complex that is essential for adherence, twitching motility, and natural competence. Although PilE is co-transcribed with these core minor pilins, it was not predicted to be included in the minor pilin complex and only modestly promoted natural transformation, whereas the other pilins were essential for this process. This work demonstrated that ComP, KK03_01180, and PilA2 are minor pilins that are dispensable for adherence and twitching motility but are important for promoting natural transformation. The similarities between phenotypes of the Δ*fimTpilVWXE* and Δ*pilC1pilC2* mutants and the AlphaFold-predicted structure lead us to hypothesize that the core minor pilins form a tip complex that loads the PilC1 and PilC2 adhesins onto the pilus fiber or stabilizes PilC1 and PilC2 on the fiber. However, in contrast to other T4P systems, this minor pilin tip complex does not appear to be required for priming of pilus assembly.

In this study, we demonstrated that elimination of each of the core minor pilins encoded by the *fimTpilVWXE* locus resulted in a significant decrease in surface piliation, suggesting that they have an important role in pilus biogenesis. Chromosomal complementation of the Δ*pilV* deletion did not increase piliation levels but significantly increased adherence, twitching motility, and transformation efficiency, providing another example where piliation level alone does not correlate with improved performance of T4P-mediated phenotypes. The roles of core minor pilins have been previously documented in other T4P-expressing bacteria. In *P. aeruginosa* and *M. xanthus*, the core minor pilins form a complex at the base of the pilus machinery that is required to prime pilus assembly (29, 30). In *K. kingae*, we identified a locus of five minor pilins (*fimTpilVWXE*) that shares some similarity to the loci encoding the *P. aeruginosa* and *M. xanthus* core minor pilins. While important for promoting high levels of surface piliation, the *K. kingae* core minor pilins were not required for pilus biogenesis, suggesting a functional divergence from *P. aeruginosa* and *M. xanthus*. In *N. gonorrhoeae*, the core minor pilins PilHIJKL show a similar phenotype where elimination of all or just one of these pilins results in low levels of surface piliation (54). In *Escherichia coli* K-12, heterologous expression of type II secretion system (T2SS) pseudopilins can compensate for core minor pilins in initiating major pilin assembly to a limited extent (55). However, our genome analysis did not identify any predicted T2SS pseudopilins in *K. kingae*. Thus, the mechanism by which *K. kingae* assembles T4P in the absence of core minor pilins remains unresolved.

Beyond the role in pilus assembly, minor pilins in other species directly mediate adherence to host cells. For instance, PilV in *N. meningitidis* is incorporated throughout the pilus and binds endothelial cells (31). In our previous studies, a highly piliated strain lacking PilC1 and PilC2 (Δ*pilC1pilC2pilT*) was completely deficient in adherence to epithelial cells, suggesting a lack of additional pilus-associated adhesins in *K. kingae* (27). The findings of this study support this prediction, given that deletion of *comP*, *pilA2*, and *KK03_01180* had no impact on adherence. In contrast, deletion of the *fimTpilVWXE* locus abrogated adherence, suggesting that the core minor pilins promote binding to epithelial cells, either directly or indirectly. Given that the Δ*fimTpilVWXE* mutant phenocopies the Δ*pilC1pilC2* mutant in piliation, adherence, and twitching motility assays, we hypothesize that FimT, PilV, PilW, PilX, and PilE indirectly promote adherence by anchoring PilC1 and PilC2 onto the pilus. This possibility aligns with findings in *P. aeruginosa* and *M. xanthus,* where core minor pilins are predicted to form a tip complex required for PilY1 association with T4P (29, 30).

The AlphaFold multimer predicted structure shown in Fig. 5 supports the prediction that the core minor pilins form a tip complex. The bulky C-terminal globular domains of PilV, PilW, and PilX cap the core channel (Fig. 5D, top view) formed by the interactions of the N-terminal alpha helices. Pilins with larger C-terminal domains than the major pilin are thought to be excluded from the pilus shaft due to steric hindrance (12). Taken together, the results of the BACTH assay and AlphaFold multimer modeling suggest that the larger FimTPilVWX minor pilins may form a complex that is separated from the bulk of the pilus fiber, which is predominantly made up of PilA1, and is intercalated with PilE, PilA2, ComP, and KK03_01180. However, AlphaFold prediction software has notable limitations, including the lack of accounting for post-translational modifications and membrane context, relevant considerations for T4P structures (56). Therefore, further studies using crystallography or cryo-electron tomography will be necessary to elucidate the structure of *K. kingae* minor pilins within T4P.

Twitching motility relies on rapid extension and retraction of T4P to pull the bacteria along a solid surface. This work demonstrated that FimT, PilV, PilW, PilX, and PilE are all individually required for twitching motility. However, chromosomal complementation of PilX and PilW did not successfully restore twitching motility. We predict that there may be stoichiometric requirements for PilX and PilW for proper T4P function and that their over- or underexpression in the complemented strains resulted in impaired twitching motility. Previous work in *N. gonorrhoeae* and *P. aeruginosa* has highlighted the importance of stoichiometric expression and incorporation of core minor pilins into T4P, demonstrating that their overexpression can result in loss of pilus formation and twitching motility (54, 57). We found that elimination of the PilT retraction ATPase in the core minor pilin mutant (Δ*fimTpilVWXEpilT*) significantly increased surface piliation, suggesting that the core minor pilins counteract pilus retraction. This phenotype is broadly observed for the PilC/PilY family (43, 58). Likely due to disrupted extension/retraction dynamics, both Δ*fimTpilVWXE* and Δ*pilC1pilC2* showed negligible twitching motility, reinforcing that PilC1, PilC2, and the core minor pilins act in concert.

In *N. meningitidis* and *N. gonorrhoeae*, the minor pilin ComP directly participates in natural competence by binding exogenous DNA (32, 35, 38). Our data suggest that *K. kingae* ComP is required for natural transformation. In pathogenic *Neisseria* species, DNA uptake relies on short, species-specific DNA uptake sequences (DUSs) (59). Previous studies identified thousands of copies of a DUS in the *K. kingae* genome called king3DUS, and a closely related *Kingella* species called *Kingella oralis* specifically bound to DNA containing king3DUS at significantly higher levels than control DNA (59). Further work is required to determine whether *K. kingae* ComP directly binds DNA and whether transformation depends on a specific DUS.

We also found that FimT, PilV, PilW, and PilX are required for natural transformation, while the Δ*pilE* mutant retains competence at levels comparable to the Δ*pilC1pilC2* mutant. These results could indicate that PilE partially contributes to ComP incorporation into the pilus or that changes in pilus extension/retraction dynamics in the Δ*pilE* mutant significantly inhibit its natural transformation efficiency. Future studies are required to understand the mechanism by which PilC1, PilC2, ComP, and the remaining minor pilins coordinate to achieve high levels of natural competence in *K. kingae*.

Minor pilins are attractive therapeutic targets because they are surface exposed, conserved across clinical isolates, and essential for pilus function in *K. kingae*. Our finding that deletion of the core minor pilins phenocopies a PilC1/PilC2 mutant suggests that targeting the FimTPilVWX complex may disrupt virulence properties, including adherence, twitching motility, and DNA uptake. Structural resolution of the minor pilin complex and a mechanistic understanding of the PilC anchoring system will be key to guiding the design of such therapeutics. Conservation of the core minor pilin locus among PilC/PilY-expressing pathogens, including *P. aeruginosa* and pathogenic *Neisseria* species, highlights the potential for broad-spectrum anti-T4P therapeutics that could inhibit the ability of pathogens to colonize and infect host niches.

## MATERIALS AND METHODS

### Bacterial strains and culture conditions

*K. kingae* strains were grown on chocolate agar at 37°C supplemented with 5% CO_2_ for ~20 hours. *E. coli* strains were cultured on LB agar at 37°C or in LB broth with shaking at 250 rpm at 37°C with 50–100 μg/mL ampicillin or 50 µg/mL kanamycin, as appropriate. All strains used in this study are listed in Table S2. *K. kingae* strains were stored at −80°C in brain heart infusion (BHI) broth containing 20% glycerol, and *E. coli* strains were stored at −80°C in LB broth containing 15% glycerol.

### Molecular biology and strain construction

For molecular cloning, DNA fragments were PCR amplified from *K. kingae* genomes or *E. coli* constructs using Q5 HiFi Master Mix (New England Biolabs, Ipswich, MA). All restriction enzymes and NEBuilder HiFi Assembly Master Mix were sourced from New England Biolabs. All plasmid constructs were confirmed to be correct through a combination of restriction digest, PCR, and sequencing using Sanger sequencing or long-read full plasmid sequencing by Plasmidsaurus.

#### Generation of in-frame *comP*, *01180*, *fimT*, *pilV*, *pilW*, *pilX*, and *pilE* mutants and complements

All primers used for the generation of mutants and complement strains are listed in Table S3. To generate the Δ*fimTpilVWXE* and Δ*comP* mutants, three fragment NEBuilder HiFi assemblies were designed using NEBuilder, which contained ~1,000 bp of the KK03 genome directly upstream of the deleted gene(s), the aphA3 cassette, and ~1,000 bp of the KK03 genome directly downstream of the stop codon in the open reading frame (ORF). All three fragments were ligated into a HindIII/EcoRI-digested pUC19 vector using NEBuilder HiFi Assembly Master Mix. The Gibson assembly product was transformed into DH5α via electroporation and plated onto selective LB agar with kanamycin (50 µg/mL). Correct constructs were linearized with NdeI and transformed into KK03 as previously described (13). To generate a complement of the Δ*fimTpilVWXE* mutant, we used pComp-Erm, which has been used to complement gene deletions in *K. kingae* previously (13, 50). The *fimTpilVWXE* operon, including its native promoter, was amplified from strain KK03 and ligated into XbaI/BamHI-digested pComp-Erm. The NEBuilder HiFi Assembly product was transformed into DH5α via electroporation and plated onto selective LB agar with ampicillin (100 µg/mL). Correct constructs were linearized with NdeI and transformed into the Δ*fimTpilVWXE K. kingae* strains as previously described (13).

To generate *01180*, *fimT*, *pilV*, *pilW*, *pilX*, and *pilE* in-frame deletions, three fragment NEBuilder HiFi assemblies were designed using NEBuilder, which contained ~1,000 bp of the KK03 genome directly upstream of the deleted gene, the *ermC* erythromycin resistance gene, and ~1,000 bp of the KK03 genome directly downstream of the stop codon. The three fragments were ligated into a HindIII/EcoRI-digested pUC19 vector using NEBuilder HiFi Assembly Master Mix. The resulting construct contained the ORF of interest replaced with the *ermC* ORF. The assembly product was transformed into DH5α via electroporation and plated onto selective LB agar with ampicillin (100 µg/mL). Correct constructs were linearized with NdeI and transformed into KK03 as previously described (13). To generate complements of Δ*01180*, Δ*fimT*, Δ*pilV*, Δ*pilW*, Δ*pilX*, and Δ*pilE*, we used pComp-Kan, which is a derivative of pComp-Erm with a kanamycin resistance cassette replacing the erythromycin cassette (13). To complement Δ*01180* and Δ*fimT*, the *fimT* and *01180* ORFs with their native promoters were amplified from strain KK03 and ligated into XbaI/BamHI or SalI/KpnI-digested pComp-Kan, respectively. To complement Δ*pilV*, Δ*pilW*, Δ*pilX*, and Δ*pilE*, two fragment NEBuilder HiFi assemblies were designed to include the native *fimT* promoter and the *pilV*, *pilW*, *pilX*, or *pilE* ORFs. The fragments were ligated into XbaI/BamHI-digested pComp-Kan. All pComp-Kan complement assemblies were transformed into DH5α via electroporation and plated onto selective LB agar with kanamycin (50 µg/mL). Correct constructs were linearized with NdeI and transformed into *K. kingae* strains as previously described (13).

To generate a complement of the Δ*pilA2* mutant, a two-fragment NEBuilder HiFi assembly was designed to insert the *pilA2* ORF downstream of the *pilA1* promoter in the pComp*_pilA1_* plasmid, which has been used to complement gene deletions in *K. kingae* previously (60). The *pilA2* ORF was ligated into XbaI/KpnI-digested pComp*_pilA1_* and transformed into DH5α via electroporation and plated onto selective LB agar with ampicillin (100 µg/mL). Correct constructs were linearized with NdeI and transformed into the Δ*pilA2 K. kingae* strain as previously described (13).

#### Generation of T18- and T25-fusion constructs for bacterial two-hybrid analysis

All primers used in the generation of BACTH constructs are listed in Table S3. The full-length mature sequences for each pilin were predicted using SignalP 6.0 software (61). To generate T18 fusions with each pilin, the mature pilin sequence was amplified from the KK03 genome and ligated into KpnI/BamHI-digested pUT18C vector using NEBuilder HiFi Assembly Master Mix. The assembly product was transformed into DH5α via electroporation and plated onto selective LB agar with ampicillin (50 µg/mL). To generate T25 fusions with each pilin, the mature pilin sequence was amplified from the KK03 genome and ligated into the KpnI/BamHI-digested pKT25 vector using NEBuilder HiFi Assembly Master Mix. The assembly product was transformed into DH5α via electroporation and plated onto selective LB agar with kanamycin (50 µg/mL).

### Eukaryotic cell lines

Chang cells (Wong-Kilbourne [D] of Chang conjunctiva, HeLa origin; American Type Culture Collection CCL-20.2) were cultivated in Modified Eagle’s Medium (MEM) supplemented with 10% fetal bovine serum and 1% non-essential amino acids, as previously described (7).

### Mass spectrometry analysis of pili

Ultrapure pili were prepared by growing *K. kingae* strains overnight on chocolate agar and suspending in phosphate-buffered saline (PBS). Samples were then homogenized at max speed for 2 min and centrifuged at 10,000 × *g* for 10 min to pellet bacteria. The bacteria-free supernatant was filtered through a 0.45 µm syringe filter and centrifuged at 50,000 × *g* for 90 min to pellet membrane fragments. The supernatant was then subjected to 20% ammonium sulfate precipitation on ice for 2 hours, and precipitated pili were collected via centrifugation at 20,000 × *g* for 20 min. Pilus preparations were resuspended in PBS and stored at −80°C for mass spectrometry analysis.

In collaboration with the Children’s Hospital of Philadelphia Proteomics Core, purified pili were solubilized and digested in-solution using the PreOmics iST kit (PreOmics GmbH, Martinsried, Germany) per the manufacturer’s protocol (62). The resulting peptides were solubilized, reduced, and alkylated by the addition of sodium deoxycholate buffer containing Tris(2-carboxyethyl)phosphine and 2-chloroacetamide. The sample was then digested for 1.5 hours at 37°C by addition of LysC and trypsin and dried via vacuum centrifugation before reconstitution in 0.1% trifluoroacetic acid (TFA) containing iRT peptides (Biognosys Schlieren, Switzerland). Peptides were analyzed on an Orbitrap Exploris 480 mass spectrometer (Thermo Fisher Scientific, San Jose, CA) coupled with an UltiMate 3000 nano UPLC System and an EasySpray source using data-dependent acquisition. Raw data were searched using MaxQuant (v.1.6.14.0) (63). The files were searched against a whole genome FASTA database of KK03, in addition to a reference database from UniProt (UP000004207) appended with sequences for common protein contaminants. The enzyme was specified as trypsin/P with a maximum of two missed cleavages. Carbamidomethylation of cysteine was specified as a fixed modification, and protein N-terminal acetylation and oxidation of methionine were considered variable modifications. A false discovery rate limit of 1% was set for peptide and protein identification. Remaining search parameters were kept as default values.

### Pilus preparations

Purification of pili from *K. kingae* was performed as previously described (27). *K. kingae* strains were grown overnight on chocolate agar plates and growth was resuspended to an OD_600_ = 0.8 in PBS. Samples were then vortexed at max speed for 3 min and centrifuged at 4,000 × *g* for 30 min to collect bacteria. The bacteria-free supernatant containing the sheared pili was filtered through a 0.45 µm syringe filter and subjected to 20% ammonium sulfate precipitation on ice for 2 hours. The precipitated pili were collected via centrifugation at 20,000 × *g* for 20 min, resuspended in PBS, and stored at −20°C for SDS-PAGE and Western blot analyses.

### SDS-PAGE, Coomassie staining, and Western blot analyses

Pilus preparations and sonicated *K. kingae* cell lysates were boiled for 5 min and separated using SDS-PAGE. For Coomassie staining, the gels were stained in R-250 Coomassie stain for 30 min prior to destaining. For Western blotting, the gels were transferred to nitrocellulose, blocked with 5% milk in PBS, and probed with an anti-pilA1 antiserum (GP-65) or a loading control GAPDH antiserum (GP-22) (27). After washing, an anti-guinea pig-horseradish peroxidase secondary antibody (1:5,000) was added, and the Western blot was developed with a chemiluminescent peroxidase substrate. The blot and gel images were captured with a G:BOX XX6 Gel Imaging System (Syngene, Cambridge UK).

### Densitometry analysis

To quantitate piliation levels, the band densities from the PilA1 Western blot on pilus preparations were measured using ImageJ software (64). For each Western blot, the PilA1 band densities from each strain were normalized to the PilA1 band density from the wild-type strain (KK03). This analysis was completed for three independent biological replicates, and the average relative PilA1 levels are presented ± standard error of the mean.

### Transmission electron microscopy

Negative-staining transmission electron microscopy was used to image type IV pili on the surface of *K. kingae* bacteria, as previously described (5, 11, 65). Briefly, *K. kingae* strains were grown on chocolate agar for ~20 hours and resuspended in 0.2 M ammonium acetate (pH 7.4). Formvar carbon-coated 300 mesh copper grids (CF300-CU, Electron Microscopy Sciences) were coated with the bacterial cell suspension for 1 min, stained for 30 seconds in UranyLess [224(23)09, Electron Microscopy Sciences], and washed in distilled water for 1 min. Grids were imaged with an FEI Tecnai T12 electron microscope.

### Quantitative bacterial adherence assays

Quantitative bacterial adherence assays were performed as described previously (11, 44, 66). Briefly, 1.8 × 10^5^ Chang epithelial cells were seeded into 24-well tissue culture-treated plates and incubated at 37°C with 5% CO_2_ for 18 hours. The cells were fixed with 2% glutaraldehyde in 0.2 M sodium phosphate buffer (pH 7.4) for 90 min at 4°C. To remove the fixative, the wells were washed four times in Tris-buffered saline (pH 7.5). *K. kingae* strains were cultured on chocolate agar for ~20 hours at 37°C with 5% CO_2_, and the bacterial growth was resuspended in BHI media to OD_600_ = 0.8. Ten microliters of the bacterial suspension was inoculated into 300 µL of pre-warmed 37°C 1× MEM in the 24-well plates containing the fixed Chang epithelial monolayers. This bacterial inoculum was plated on chocolate agar to determine the CFU per milliliter. The plates were centrifuged for 5 min at 1,000 rpm to bring the bacteria into proximity with the monolayer and incubated at 37°C with 5% CO_2_ for 25 min. To remove non-adherent bacteria, the wells were washed 4× with PBS. A volume of 100 µL of trypsin-EDTA (0.05%) was added, and the plates were incubated for 20 min at 37°C with 5% CO_2_ to facilitate recovery of adherent bacteria. The recovered bacteria were plated on chocolate agar to determine the CFU per milliliter. The percent adherence was calculated as a ratio of recovered bacteria to the inoculum.

### Twitching motility assays

Twitching motility assays were carried out as previously described (66). *K. kingae* strains were cultured on chocolate agar for ~20 hours at 37°C with 5% CO_2_, and the growth was resuspended in 1× PBS to an OD_600_ = 0.8. One microliter of the bacterial suspension was stab inoculated into the bottom of tissue culture-treated petri dishes containing chocolate agar (1% agar). Plates were incubated for 72 hours at 37°C with 5% CO_2_. After 72 hours, the chocolate agar was removed, and the zone of bacterial spread was stained with 0.1% crystal violet. Three diameters of the crystal violet-stained bacterial spread were measured and reported in millimeters.

### Natural transformation efficiency assays

Transformation efficiency assays were performed as previously described (27). *K. kingae* strains were cultured on chocolate agar for ~20 hours at 37°C, 5% CO_2_, and the growth was resuspended in BHI media to OD_600_ = 0.8. One microgram of plasmid DNA containing ~1,000 bp of upstream and downstream sequence flanking the *knh* gene, and a kanamycin, erythromycin, or tetracycline resistance cassette in place of the *knh* gene was added to 250 µL of bacterial suspension in a 24-well plate. The 24-well plate was incubated at room temperature for 30 min before 250 µL of 20% lysed horse blood in BHI was added to each well. The mixture was incubated at 37°C, 5% CO_2_, for 2.5 hours to allow for bacterial recovery before CFU plating on chocolate agar containing either kanamycin (50 µg/mL), erythromycin (1 µg/mL), or tetracycline (2 µg/mL), as appropriate. The natural competence efficiency was calculated based on the ratio of recovered bacteria to inoculum.

### Bacterial two-hybrid assays

A BACTH system (Euromedex, Souffelweyersheim, France) was used to evaluate protein-protein interactions, as previously described (47). Briefly, full-length mature pilins (PilA1, FimT, PilV, PilW, PilX, and PilE) were N-terminally tagged with the T18 or T25 fragment of CyaA using the pUT18C and pKT25 vector plasmids, respectively. T25-tagged fusion proteins and T18-tagged fusion proteins were co-transformed into the *cya* mutant strain BTH101 (F^−^, *cya-99*, *araD139*, *galE15*, *galK16*, *rpsL1* [*Str^r^*], *hsdR2*, *mcrA1*, and *mcrB1*). Three random colonies were selected and grown overnight in LB containing ampicillin (50 µg/mL) and kanamycin (50 µg/mL). The co-transformants were subcultured 1:100 in LB containing ampicillin (50 µg/mL), kanamycin (50 µg/mL), and 0.5 mM isopropyl β-D-thiogalactopyranoside (IPTG) and grown at 37°C to OD_600_ = 0.6. Five microliters of each culture was spot plated on LB agar containing 50 µg/mL ampicillin, 50 µg/mL kanamycin, 0.5 mM IPTG, and 40 µg/mL X-gal. The plates were incubated at 30°C overnight and imaged.

### Bioinformatic analyses

The Expasy ProtParam software was used to predict the molecular weight of the *K. kingae* pilins (https://web.expasy.org/protparam/) (67). Signal peptide predictions for BACTH analyses were accomplished using SignalP 6.0 (https://services.healthtech.dtu.dk/services/SignalP-6.0/) (61). Genomic sequences and primary amino acid sequences were analyzed with Nucleotide BLAST and Protein BLAST, respectively, hosted at the National Center for Biotechnology Information (www.ncbi.nlm.nih.gov) (68). Sequence similarity and identity between the minor pilins were calculated using the EMBOSS Needle Pairwise Sequence Alignment tool (https://www.ebi.ac.uk/jdispatcher/psa/emboss_needle) (69). Genomic alignments of loci encoding core minor pilins in T4P-expressing species were generated using Easyfig (https://mjsull.github.io/Easyfig/) (70). Structural prediction was accomplished using AlphaFold 3 and AlphaFold 3 multimer (www.alphafoldserver.com) (42). AlphaFold 3 structural predictions were visualized using ChimeraX 1.9-rc2024.11.01 (71).

## Data Availability

The mass spectrometry proteomics data have been deposited to the ProteomeXchange Consortium via the PRIDE (72) partner repository with the data set identifier PXD068177 and 10.6019/PXD068177. All other data are within the article and its supporting information files.

## SUPPLEMENTAL MATERIAL

Supplemental Figures and Tables mbio.02790-25-s0001.pdf Figures S1-S7, Tables S1-S3, and supporting references.

ASM does not own the copyrights to Supplemental Material that may be linked to, or accessed through, an article. The authors have granted ASM a non-exclusive, world-wide license to publish the Supplemental Material files. Please contact the corresponding author directly for reuse.
