# Supplementary material for: Multi-functional minor pilins coordinate type IV pilus assembly, adherence, motility, and DNA uptake in the pediatric pathogen Kingella kingae
Source: mBio. 2025 Oct 13;16(11):e02790-25. doi: 10.1128/mbio.02790-25 (PMC12607826; doi:10.1128/mbio.02790-25)
Supplement: Supplemental Figures and Tables — Figures S1-S7, Tables S1-S3, and supporting references. [file mbio.02790-25-s0001.pdf]

SUPPORTING INFORMATION

Multi-functional Minor Pilins Coordinate Type IV Pilus Assembly, Adherence, Motility, and DNA Uptake in the Pediatric Pathogen *Kingella kingae*

Taylor A. Yount[1,2], Eric A. Porsch[1], and Joseph W. St. Geme III[1,2]*

[1]Children's Hospital of Philadelphia, Philadelphia, Pennsylvania, USA

[2]Perelman School of Medicine, University of Pennsylvania, Philadelphia, Pennsylvania, USA

*Corresponding author: stgemeiiij@chop.edu (JWS)

Keywords: Type IV pili, minor pilins, adherence, twitching motility, natural competence

Materials included:

- Figures S1 – S7
- Tables S1 – S3
- Supporting References

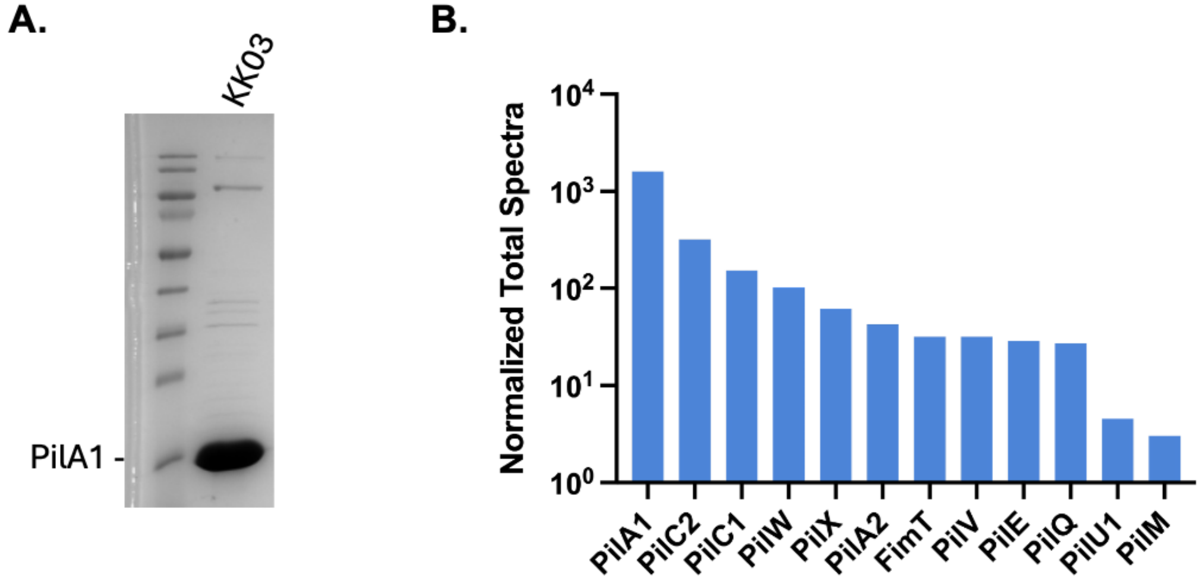

Figure S1. (A) Coomassie-stained 15% SDS-PAGE gel containing the ultrapure pili sample from KK03 that was sent for mass spectrometry analysis. **(B)** The total spectra for each T4P protein was normalized by the total number of spectra in the sample.

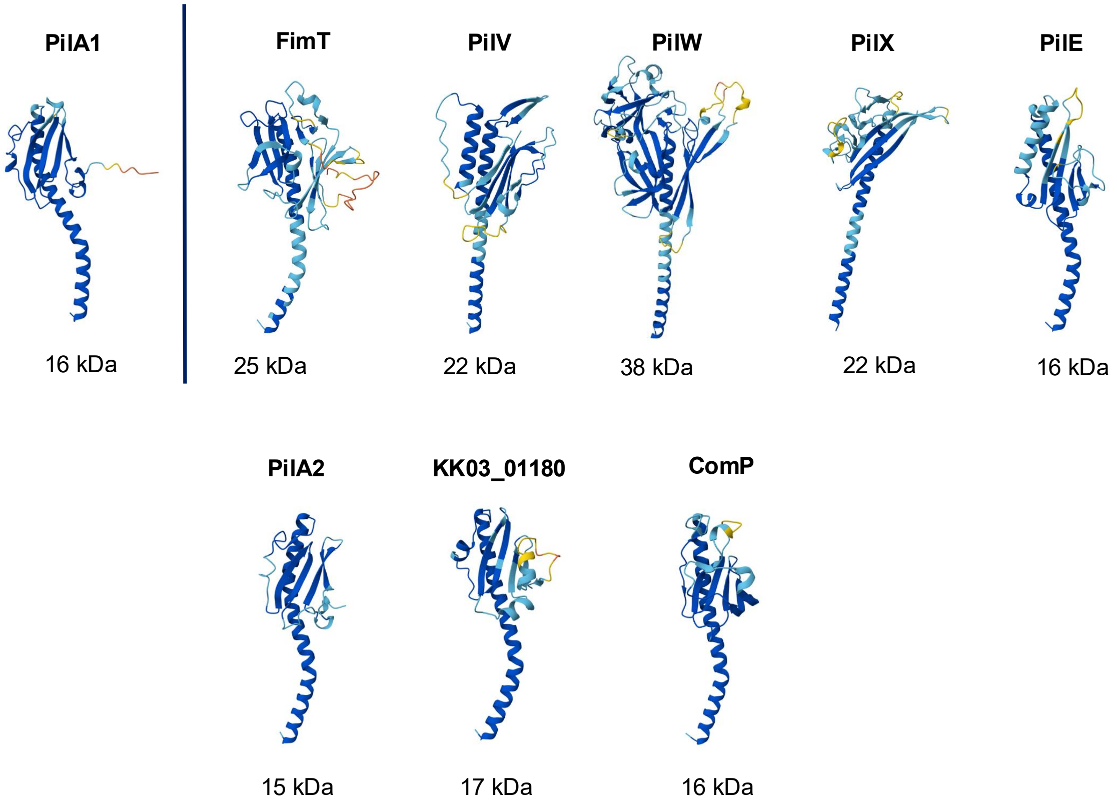

Figure S2. AlphaFold 3-predicted structures for each of the mature KK03 pilins. The confidence levels are represented by the predicted Local Distance Difference Test (pLDDT), where dark blue (pLDDT > 90) represents very high confidence, light blue (pLDDT 70-90) represents average confidence, and yellow and orange (pLDDT < 70) represent low confidence in the local structural prediction. The predicted molecular weight for each pilin is listed under the corresponding predicted structure.

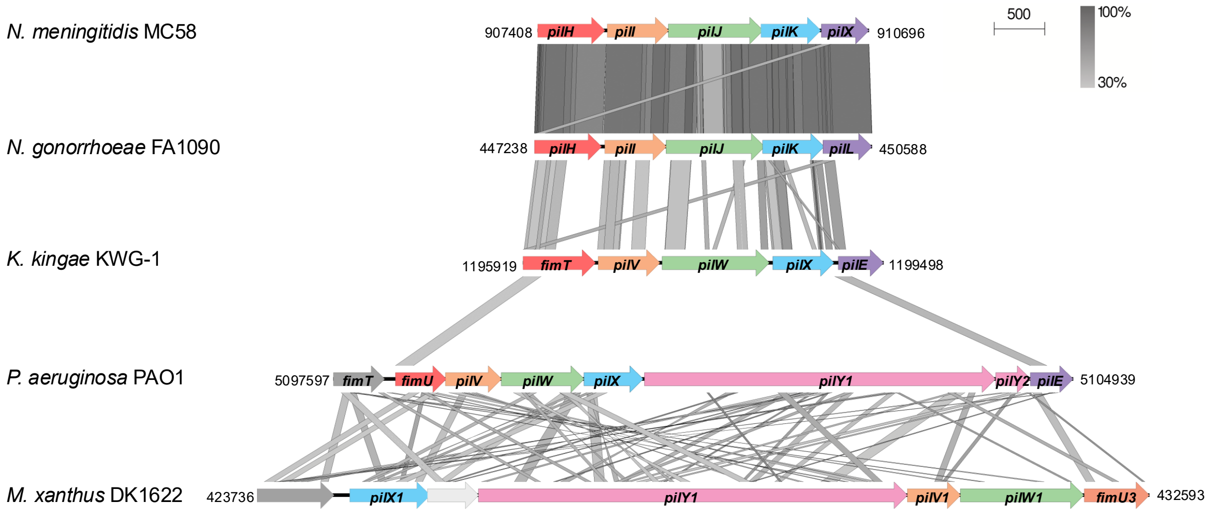

Figure S3. BLAST alignment of loci encoding core minor pilins from *N. meningitidis, N. gonorrhoeae, K. kingae, P. aeruginosa,* and *M. xanthus.* The gray gradient between the aligned loci represents the percent identity in the local sequences (top right). The loci are drawn to scale according to the scale bar which represents 500 bp (top right).

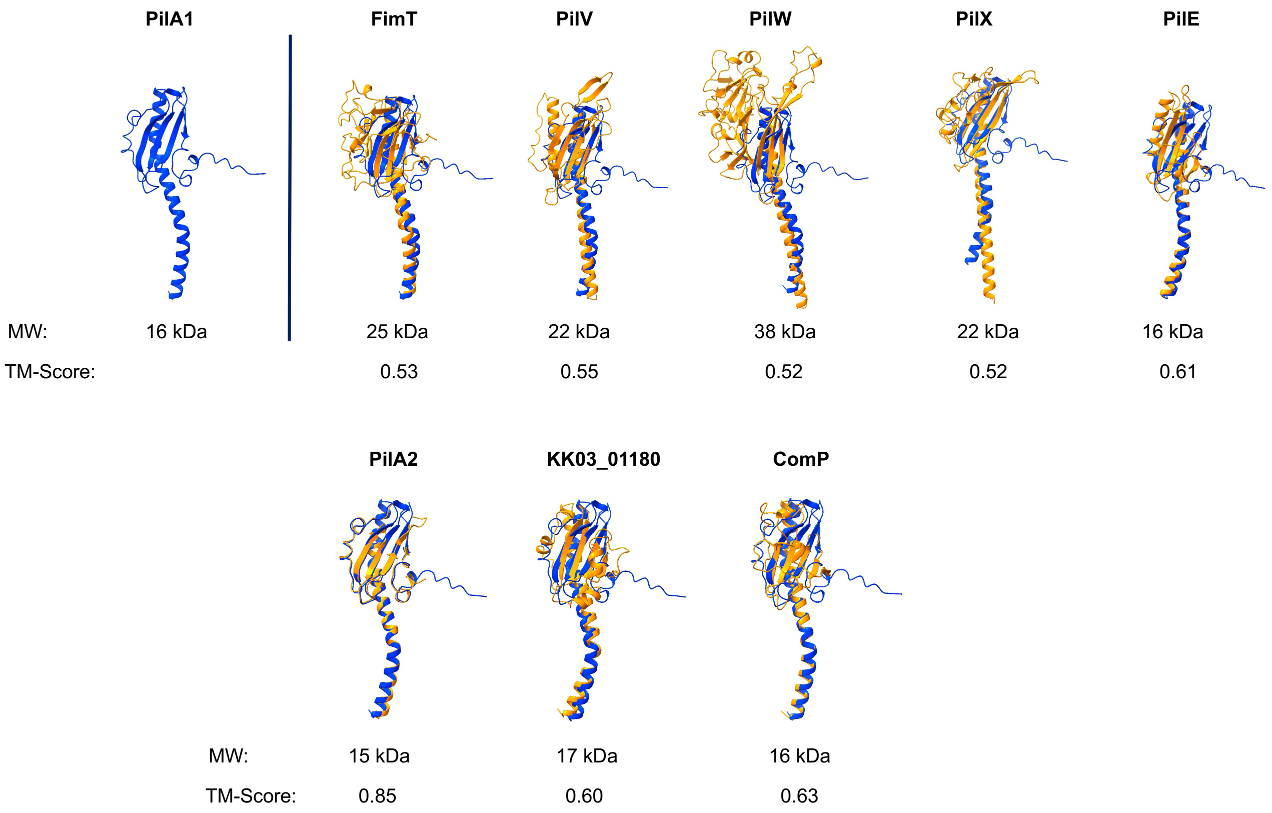

Figure S4. TM-align pairwise structure alignment between PilA1 (blue) and the minor pilins (orange). The molecular weight for each minor pilin is displayed below the superimposed structure. The template modeling score (TM-score) is a metric used to assess the topological similarity between the two protein structures. TM-scores fall within the range of 0 to 1, where 1 indicates a perfect match between the predicted structures and 0 signifies no structural similarity.

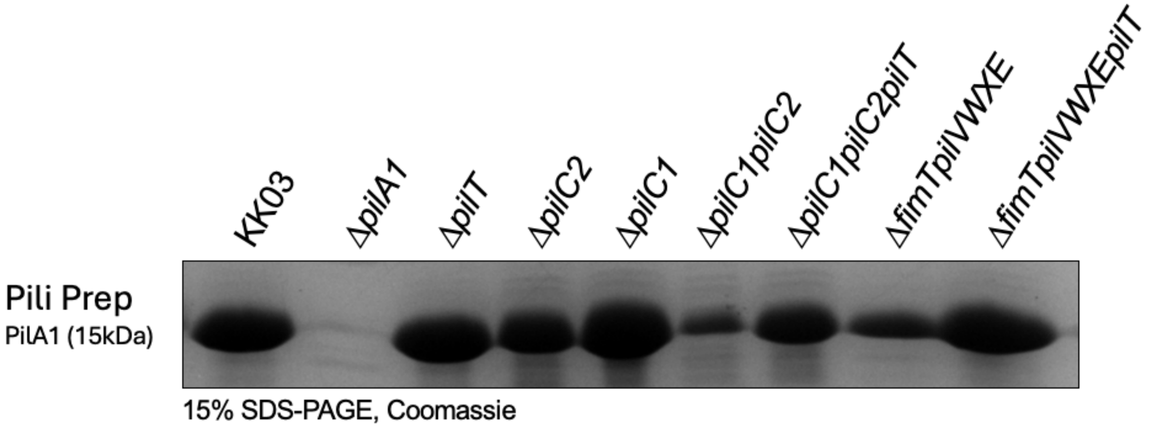

Figure S5. Purified pili from each *K. kingae* strain were boiled, separated via SDS-PAGE, and stained with Coomassie. The ~15 kDa band represents PilA1 levels in the purified pili, which is a proxy for the level of surface piliation in the corresponding strain.

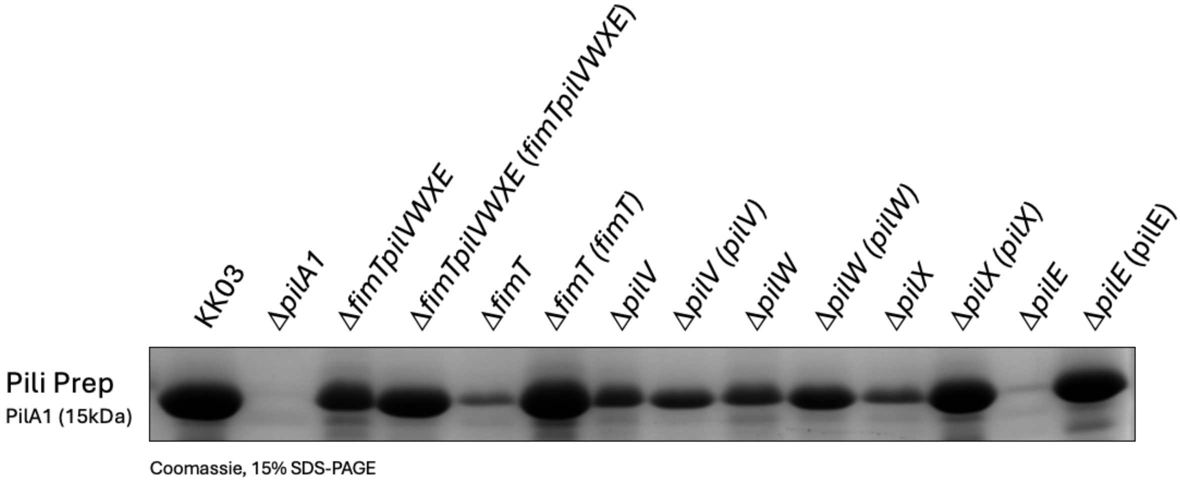

Figure S6. Purified pili from each *K. kingae* strain were boiled, separated via SDS-PAGE, and stained with Coomassie. The ~15 kDa band represents PilA1 levels in the purified pili, which is a proxy for the level of surface piliation in the corresponding strain.

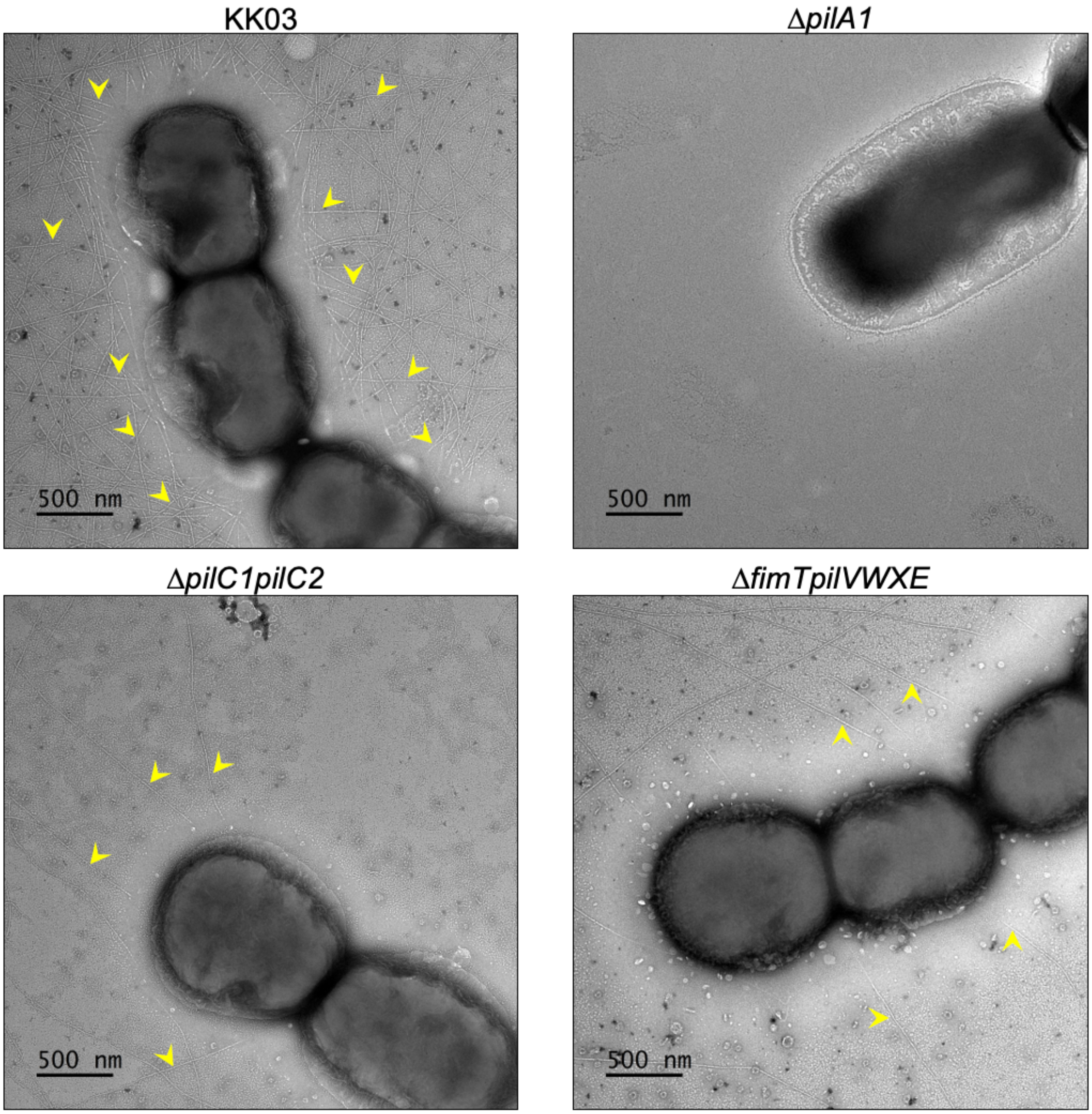

Figure S7. Negative-stained transmission electron micrographs of *K. kingae* strains. Examples of type IV pili extending from the bacteria are labeled with yellow arrows.

Table S1. Identification of pilin-like proteins in *K. kingae* type IV pili.

T4P Protein (TXSScan)	Mass Spec on Sheared Pili	Pilin-Like Structure?	Percent Identity/ Similarity to PilA1
**PilA1**	■	■	100/100
**PilA2**	■	■	50/65
FimB			☒
PilC1	■		☒
PilC2	■		☒
**FimT**	■	■	20/30
**PilV**	■	■	20/32
**PilW**	■	■	9/15
**PilX**	■	■	16/23
**PilE**	■	■	25/40
**ComP**		■	23/36
**KK03_01180**		■	27/43
PilT	■		☒
PilU1	■		☒
PilU2	■		☒
PilD			☒
PilM	■		☒
PilN			☒
PilO	■		☒
PilP			☒
PilQ	■		☒

Table S1. Type IV pilus proteins were identified using the bioinformatic tool TXSScan and mass spectrometry on pili purified from strain KK03. Dark gray boxes represent a positive identification of that protein in the mass spectrometry data. To identify pilin-like proteins (green), we used AlphaFold 3 to characterize the predicted structures of each T4P protein. Dark gray boxes represent proteins with a pilin-like AlphaFold-predicted structure. The percent identity and

similarities to the major pilin PilA1 were calculated using the EMBOSS Needle Pairwise Sequence Alignment tool.

Table S2. Bacterial strains and plasmids used in this study.

Strain or Plasmid	Description	Source
K. kingae		
KK01	Nonspreading/noncorroding derivative of clinical isolate 269–492	(1)
KK03	Spreading/corroding derivative of clinical isolate 269-492	(1)
KK03Δ*pilA1*	KK03 with an *aphA3* marked *pilA1* deletion	(2)
KK03Δ*pilC1pilC2*	KK03 with a *tetM* marked *pilC1* deletion and an unmarked *pilC2* deletion	(3)
KK03Δ*pilA2*	KK03 with an *aphA3* marked *pilA2* deletion	(2)
KK03Δ*comP*	KK03 with an *aphA3* marked *comP* deletion	This study
KK03Δ*01180*	KK03 with an *ermC* marked *KK03_01180*	This study
KK03Δ*fimTpilVWXE*	KK03 with an *aphA3* marked *fimTpilVWXE* deletion	This study
KK03Δ*fimT*	KK03 with an *ermC* marked *fimT* deletion	This study
KK03Δ*pilV*	KK03 with an *ermC* marked *pilV* deletion	This study
KK03Δ*pilW*	KK03 with an *ermC* marked *pilW* deletion	This study
KK03Δ*pilX*	KK03 with an *ermC* marked *pilX* deletion	This study
KK03Δ*pilE*	KK03 with an *ermC* marked *pilE* deletion	This study
KK03Δ*fimTpilVWXE* (*fimTpilVWXE*)	KK03Δ*fimTpilVWXE* with *fimTpilVWXE* complemented at an unlinked locus under control of the *fimT* promoter	This study
KK03Δ*fimT* (*fimT*)	KK03Δ*fimT* with *fimT* complemented at an unlinked locus under control of the *fimT* promoter	This study
KK03Δ*pilV* (*pilV*)	KK03Δ*pilV* with *pilV* complemented at an unlinked locus under control of the *fimT* promoter	This study
KK03Δ*pilW* (*pilW*)	KK03Δ*pilW* with *pilW* complemented at an unlinked locus under control of the *fimT* promoter	This study
KK03Δ*pilX* (*pilX*)	KK03Δ*pilX* with *pilX* complemented at an unlinked locus under control of the *fimT* promoter	This study
KK03Δ*pilE* (*pilE*)	KK03Δ*pilE* with *pilE* complemented at an unlinked locus under control of the *fimT* promoter	This study
KK03Δ*pilA2* (*pilA2*)	KK03Δ*pilA2* with *pilA2* complemented at an unlinked locus under control of the *pilA1* promoter	This study
KK03Δ*01180* (*01180*)	KK03Δ*01180* with *KK03_01180* complemented at an unlinked locus under control of the *KK03_01180* promoter	This study
KK03Δ*pilT*	KK03 with an ermC marked *pilT* deletion	(4)
E. coli		
BTH101	F^-, *cya-99, araD139, galE15, galK16, rpsL1 (Str* ^{r}*), hsdR2, mcrA1, mcrB1.*	Euromedex

DH5α	$\lambda^{-}\phi$80d*lac*ZΔM15 Δ(*lacZYA-argF*)*U169 recA1 endA1* hsdR17(r_K^{-} m_K^{-}) *supE44 thi-1 gyrA relA1*	Thermo Fisher Scientific
Plasmids		
pUC19	High copy number cloning vector	(5)
pUC19/Δ*comP*:kan	*comP* deletion construct with an *aphA3* marked *comP* deletion	This study
pUC19/Δ*fimTpilVWXE*:kan	*fimTpilVWXE* deletion construct with an *aphA3* marked *fimTpilVWXE* deletion	This study
pUC19/Δ*01180*:erm	*01180* deletion construct with an *ermC* marked *01180* deletion	This study
pUC19/Δ*fimT*:erm	*fimT* deletion construct with an *ermC* marked *fimT* deletion	This study
pUC19/Δ*pilV*:erm	*pilV* deletion construct with an *ermC* marked *pilV* deletion	This study
pUC19/Δ*pilW*:erm	*pilW* deletion construct with an *ermC* marked *pilW* deletion	This study
pUC19/Δ*pilX*:erm	*pilX* deletion construct with an *ermC* marked *pilX* deletion	This study
pUC19/Δ*pilE*:erm	*pilE* deletion construct with an *ermC* marked *pilE* deletion	This study
pFalcon2	Source of the *aphA3* kanamycin resistance cassette	(6)
pIDN4	Source of *ermC* erythromycin resistance cassette	(7)
pComp-Erm	Complementation plasmid for introducing *ermC*-marked (erythromycin) chromosomal complements into *K. kingae*	(8)
$pComp_{pilA1}$	pComp-Erm containing the *K. kingae pilA1* promoter to drive expression of a complemented gene	(9)
pComp-*fimTpilVWXE*	pComp-Erm containing *fimTpilVWXE* for complementation under control of the *fimT* promoter	This study
$pComp_{pilA1}$-*pilA2*	pComp-Erm containing *pilA2* for complementation under control of the *pilA1* promoter	This study
pComp-Kan	Complementation plasmid for introducing *aphA3*-marked (kanamycin) chromosomal complements into *K. kingae*	(8)
pComp-*01180*	pComp-Kan containing *01180* for complementation under control of the *01180* promoter	This study
pComp-*fimT*	pComp-Kan containing *fimT* for complementation under control of the *fimT* promoter	This study
pComp-*pilV*	pComp-Kan containing *pilV* for complementation under control of the *fimT* promoter	This study
pComp-*pilW*	pComp-Kan containing *pilW* for complementation under control of the *fimT* promoter	This study
pComp-*pilX*	pComp-Kan containing *pilX* for complementation under control of the *fimT* promoter	This study

pComp-*pilE*	pComp-Kan containing *pilE* for complementation under control of the *fimT* promoter	This study
pUT18C	Plasmid that encodes the T18 fragment (amino acids 225 to 399) of CyaA and a multicloning site inserted to allow construction of in-frame fusions at the C-terminal end of the T18 polypeptide. Derived from a high copy number vector pUC19, expressing an ampicillin resistant marker.	Euromedex
pUT18C-zip	pUT18C construct with an in-frame fusion of the T18 fragment with the N-terminus of the leucine zipper of GCN4.	(10)
pUT18C-PilA1	pUT18C construct with an in-frame fusion of the T18 fragment with the N-terminus of the mature, full-length PilA1 pilin.	This study
pUT18C-FimT	pUT18C construct with an in-frame fusion of the T18 fragment with the N-terminus of the mature, full-length FimT pilin.	This study
pUT18C-PilV	pUT18C construct with an in-frame fusion of the T18 fragment with the N-terminus of the mature, full-length PilV pilin.	This study
pUT18C-PilW	pUT18C construct with an in-frame fusion of the T18 fragment with the N-terminus of the mature, full-length PilW pilin.	This study
pUT18C-PilX	pUT18C construct with an in-frame fusion of the T18 fragment with the N-terminus of the mature, full-length PilX pilin.	This study
pUT18C-PilE	pUT18C construct with an in-frame fusion of the T18 fragment with the N-terminus of the mature, full-length PilE pilin.	This study
pUT18C-PilA2	pUT18C construct with an in-frame fusion of the T18 fragment with the N-terminus of the mature, full-length PilA2 pilin.	This study
pUT18C-ComP	pUT18C construct with an in-frame fusion of the T18 fragment with the N-terminus of the mature, full-length ComP pilin.	This study
pUT18C-01180	pUT18C construct with an in-frame fusion of the T18 fragment with the N-terminus of the mature, full-length KK03_01180 pilin.	This study
pKT25	Plasmid that encodes the T25 fragment (first 224 amino acids) of CyaA and a multicloning site inserted to allow construction of in-frame fusions at the C-terminal end of the T25 polypeptide. Derived from a low copy-number plasmid pSU40, expressing a kanamycin resistant marker.	Euromedex

pKT25-zip	pKT25 construct with an in-frame fusion of the T25 fragment with the N-terminus of the leucine zipper of GCN4.	(10)
pKT25-PilA1	pKT25 construct with an in-frame fusion of the T25 fragment with the N-terminus of the mature, full-length PilA1 pilin.	This study
pKT25-FimT	pKT25 construct with an in-frame fusion of the T25 fragment with the N-terminus of the mature, full-length FimT pilin.	This study
pKT25-PilV	pKT25 construct with an in-frame fusion of the T25 fragment with the N-terminus of the mature, full-length PilV pilin.	This study
pKT25-PilW	pKT25 construct with an in-frame fusion of the T25 fragment with the N-terminus of the mature, full-length PilW pilin.	This study
pKT25-PilX	pKT25 construct with an in-frame fusion of the T25 fragment with the N-terminus of the mature, full-length PilX pilin.	This study
pKT25-PilE	pKT25 construct with an in-frame fusion of the T25 fragment with the N-terminus of the mature, full-length PilE pilin.	This study
pKT25-PilA2	pKT25 construct with an in-frame fusion of the T25 fragment with the N-terminus of the mature, full-length PilA2 pilin.	This study
pKT25-ComP	pKT25 construct with an in-frame fusion of the T25 fragment with the N-terminus of the mature, full-length ComP pilin.	This study
pKT25-01180	pKT25 construct with an in-frame fusion of the T25 fragment with the N-terminus of the mature, full-length KK03_01180 pilin.	This study

Table S3. Primers used in this study.

Primer name	Sequence (5'→3')
comPupF	CGACGTTGTAAAACGACGGCCAGTGACGGCGATGTATCGGGAATTG
comPupR	TAAGCTGTCAAACACACTTCCATTAACGTGAATCCAG
comPkanF	TAATGGAAGTGTGTTTGACAGCTTATCATCG
comPkanR	ATATTACGGATACATCTAAATCTAGGTACTAAAACAATTC
comPdownF	CTAGATTTAGATGTATCCGTAATATTAAAAAGCAGC
comPdownR	AACAGCTATGACCATGATTACGCCATCACACCGTTCATTGTCG
01180upF	CGACGTTGTAAAACGACGGCCAGTGTTTATGAAGTTGGTAGATGG
01180upR	TCCCCAAAGCGTAGGATAAAAGTTTTCATTTTATTTAATACC
01180ermF	AAACTTTTATCCTACGCTTTGGGGAAATTATG
01180ermR	ATGCGATTAGCTGGTGTAATCATGGTCATAG
01180downF	CCATGATTACACCAGCTAATCGCATCAATAAAATCAACAAAAAG
01180downR	AACAGCTATGACCATGATTACGCCACAGAACCCGCGCAGTTTG
fimTpilVWXEupF	CGACGTTGTAAAACGACGGCCAGTGAGTTGGCAGAAGTTGGAAC
fimTpilVWXEupR	TAAGCTGTCAAACTTTTTGATATTGATTCATAGCTATTTTACAC
fimTpilVWXEkanF	CAATATCAAAAAGTTTGACAGCTTATCATCG
fimTpilVWXEkanR	AGCTGCTGGTGTTCATCTAAATCTAGGTACTAAAACAATTC
fimTpilVWXEdownF	TAGATTTAGATGAACACCAGCAGCTACGAAAC
fimTpilVWXEdownR	AACAGCTATGACCATGATTACGCCAGAATGCTTTTTGCACCTGC
fimTupF	TTGTAAAACGACGGCCAGTGGCTGCTTGATGAAGCGGAAAATC
fimTupR	TCTCGTTCATAGCTATTTTACACTTTCAAATGATTTACTTTTCTTAATC
fimTermF	TAAAATAGCTATGAACGAGAAAAATATAAAACAC
fimTermR	GTTATTATCAAATTACTTATTAAATAATTTATAGCTATTGAAAAG
fimTdownF	TAATAAGTAATTTGATAATAACAGTTAGGAAAATAAATGAAAAATATGACTTATTTTC
fimTdownR	CTATGACCATGATTACGCCAACGCACGCCATAGCCTTC
pilVupF	CGACGTTGTAAAACGACGGCCAGTGAATCAGGTGCAATCGAGC
pilVupR	TTTTCTCGTTCATTTATTTTCCTAACTGTTATTATCAAATTATGC
pilVermF	TTAGGAAAATAAATGAACGAGAAAAATATAAAACAC

pilVermR	TTCCCGTATCTATTACTTATTAAATAATTTATAGCTATTGAAAAG
pilVdownF	ATTTAATAAGTAATAGATACGGGAAATACTATAATG
pilVdownR	AACAGCTATGACCATGATTACGCCATCATCAATAGATGTACGATATTTTAAATG
pilWupF	CGACGTTGTAAAACGACGGCCAGTGCGTAAAATGGCGGTGGCAC
pilWupR	TTTTCTCGTTCATTATAGTATTTCCCGTATCTATTAGTTGCC
pilWermF	GGAAATACTATAATGAACGAGAAAAATATAAAACAC
pilWermR	TGCTGAACTAACTTACTTATTAAATAATTTATAGCTATTGAAAAG
pilWdownF	ATTTAATAAGTAAGTTAGTTCAGCAAACAAG
pilWdownR	AACAGCTATGACCATGATTACGCCACCTGCATATAGATAGACAC
pilXupF	CGACGTTGTAAAACGACGGCCAGTGAACTTATTTCATGACGCG
pilXupR	TTTTCTCGTTCATATTATTCCTTATTTACTTGTTTGC
pilXermF	ATAAGGAATAATATGAACGAGAAAAATATAAAACAC
pilXermR	CTTCATATCGTATTACTTATTAAATAATTTATAGCTATTGAAAAG
pilXdownF	ATTTAATAAGTAATACGATATGAAGATAAAAAACTCAGG
pilXdownR	AACAGCTATGACCATGATTACGCCACCAAGACCACAATCGCAC
pilEupF	CGACGTTGTAAAACGACGGCCAGTGTATGCTGGGCAGTGTAAATG
pilEupR	TTTTCTCGTTCATATCGTATTACCGTAATAATTCAAC
pilEermF	CGGTAATACGATATGAACGAGAAAAATATAAAACAC
pilEermR	TTATCCATCAATTTACTTATTAAATAATTTATAGCTATTGAAAAG
pilEdownF	ATTTAATAAGTAAATTGATGGATAAACCGAATTTTG
pilEdownR	AACAGCTATGACCATGATTACGCCATGATAAACGCATCTAAATCTC
fimTpilVWXE_comp_F	GACCATGATTACACCGGTACCCGGGTGCTTTTCCCACAAAAAAACC
fimTpilVWXE_comp_R	AAGCTTGCATGCCTGCAGGTCGACTCGAACCGATAAACGCCCAATG
fimT_comp_F	GATAAGCTGTCAAACGGTACCCGGGCGAACCGATAAACGCCCAATG
fimT_comp_R	AAGCTTGCATGCCTGCAGGTCGACTTTATGCAGCTGCACAGATAC
fimTpromoterF	GCTGTCAAACGGTACCCGGGCGAACCGATAAACGCCCAATG
fimTpromoter_comp_pilV_R	ATATTTTTCATAGCTATTTTACACTTTCAAATGATTTACTTTTC
pilVcompF	TAAAATAGCTATGAAAAATATGACTTATTTTCATACTAAATATTC

pilVcompR	TGCATGCCTGCAGGTCGACTTTAGTTGCCTACTTCTAGC
fimTpromoter_comp_pilW_R	GCTTATTCATAGCTATTTTACACTTTCAAATGATTTACTTTTC
pilWcompF	TAAAATAGCTATGAATAAGCGTTTTATTTCAAAAC
pilWcompR	TGCATGCCTGCAGGTCGACTTTAAATACTTGGCGTTACTG
fimTpromoter_comp_pilX_R	TATTTTTCATAGCTATTTTACACTTTCAAATGATTTACTTTTC
pilXcompF	GTAAAATAGCTATGAAAAATATAATCTCTCGAAATC
pilXcompR	TGCATGCCTGCAGGTCGACTTTACCGTAATAATTCAACATAAC
fimTpromoter_comp_pilE_R	TTATCTTCATAGCTATTTTACACTTTCAAATGATTTACTTTTC
pilEcompF	TAAAATAGCTATGAAGATAAAAAACTCAGGATTC
pilEcompR	TGCATGCCTGCAGGTCGACTTTAAAACACTTCGCACCC
pilA2compF	TATGACCATGATTACGGTACTTAATTACGGCATTCCGATG
pilA2compR	TTATTTTTGGAGACAAAACTCTAGATGGAAACTAAAATGCAAGG
01180compF	GATAAGCTGTCAAACGGTACTAATGCTATCGGCACGAG
01180compR	CAAGCTTGCATGCCTGCAGGTGATATAGCAAAAAGTGCAG
pUT18CpilA1F	ACTGCAGGTCGACTCTAGAGTTCACATTAATCGAGTTGATG
pUT18CpilA1R	GATGAATTCGAGCTCGGTACTTAGCCACCATTGGTAGC
pUT18CfimTF	ACTGCAGGTCGACTCTAGAGTTTACTCTGATTGAGTTGATGG
pUT18CfimTR	GATGAATTCGAGCTCGGTACTTATGCAGCTGCACAGATAC
pUT18CpilVF	ACTGCAGGTCGACTCTAGAGGCAACACTACTGGAAGTG
pUT18CpilVR	GATGAATTCGAGCTCGGTACTTAGTTGCCTACTTCTAGC
pUT18CpilWF	ACTGCAGGTCGACTCTAGAGTTTACTTTAATCGAATTTTTGGTTG
pUT18CpilWR	GATGAATTCGAGCTCGGTACTTAAATACTTGGCGTTACTG
pUT18CpilXF	ACTGCAGGTCGACTCTAGAGTTTCTTTGTTTTTTGTGATGATTTTAATG
pUT18CpilXR	GATGAATTCGAGCTCGGTACTTACCGTAATAATTCAACATAAC
pUT18CpilEF	ACTGCAGGTCGACTCTAGAGTTCACATTGGTTGAAATGATG
pUT18CpilER	GATGAATTCGAGCTCGGTACTTAAAACACTTCGCACCC
pUT18CpilA2F	ACGCCACTGCAGGTCGACTCTAGAGTTTACCTTAATTGAATTGATGATTG
pUT18CpilA2R	ATATCGATGAATTCGAGCTCGGTACTTAATTACGGCATTCCGATG
pUT18CcomPF	ACGCCACTGCAGGTCGACTCTAGAGTTCACGTTAATGGAAGTGTTG
pUT18CcomPR	ATATCGATGAATTCGAGCTCGGTACCGGATACGCTCGACAATTTTTATC

pUT18C01180F	ACGCCACTGCAGGTCGACTCTAGAGTTTACCTTGGTTGAACTTATG
pUT18C01180R	ATATCGATGAATTCGAGCTCGGTACTTAGCTTAATGGTTCGCAG
pKT25pilA1F	CTGCAGGGTCGACTCTAGAGTTCACATTAATCGAGTTGATG
pKT25pilA1R	ATTCTTAGTTACTTAGGTACTTAGCCACCATTGGTAGC
pKT25fimTF	CTGCAGGGTCGACTCTAGAGTTTACTCTGATTGAGTTGATGG
pKT25fimTR	ATTCTTAGTTACTTAGGTACTTATGCAGCTGCACAGATAC
pKT25pilVF	CTGCAGGGTCGACTCTAGAGGCAACACTACTGGAAGTG
pKT25pilVR	ATTCTTAGTTACTTAGGTACTTAGTTGCCTACTTCTAGC
pKT25pilWF	CTGCAGGGTCGACTCTAGAGTTTACTTTAATCGAATTTTTGGTTG
pKT25pilWR	ATTCTTAGTTACTTAGGTACTTAAATACTTGGCGTTACTG
pKT25pilXF	CTGCAGGGTCGACTCTAGAGTTTTCTTTGTTTTTTGTGATGATTTTAATG
pKT25pilXR	ATTCTTAGTTACTTAGGTACTTACCGTAATAATTCAACATAAC
pKT25pilEF	CTGCAGGGTCGACTCTAGAGTTCACATTGGTTGAAATGATG
pKT25pilER	ATTCTTAGTTACTTAGGTACTTAAAACACTTCGCACCC
pKT25pilA2F	GCGGGCTGCAGGGTCGACTCTAGAGTTTACCTTAATTGAATTGATGATTG
pKT25pilA2R	GCCGAATTCTTAGTTACTTAGGTACTTAATTACGGCATTCCGATG
pKT25comPF	GCGGGCTGCAGGGTCGACTCTAGAGTTCACGTTAATGGAAGTGTTG
pKT25comPR	GCCGAATTCTTAGTTACTTAGGTACTTACGGATACGCTCGACAATTTTTATC
pKT2501180F	GCGGGCTGCAGGGTCGACTCTAGAGTTTACCTTGGTTGAACTTATG
pKT2501180R	GCCGAATTCTTAGTTACTTAGGTACTTAGCTTAATGGTTCGCAG

SUPPORTING REFERENCES

1. Kehl-Fie TE, St. Geme JW. 2007. Identification and Characterization of an RTX Toxin in the Emerging Pathogen *Kingella kingae*. J Bacteriol 189:430–436.

2. Kehl-Fie TE, Miller SE, St. Geme JW. 2008. *Kingella kingae* Expresses Type IV Pili That Mediate Adherence to Respiratory Epithelial and Synovial Cells. JB 190:7157–7163.

3. Sacharok AL, Porsch EA, Yount TA, Keenan O, St. Geme JW. 2022. *Kingella kingae* PilC1 and PilC2 are adhesive multifunctional proteins that promote bacterial adherence, twitching motility, DNA transformation, and pilus biogenesis. PLoS Pathog 18:e1010440.

4. Porsch EA, Johnson MDL, Broadnax AD, Garrett CK, Redinbo MR, St. Geme JW. 2013. Calcium Binding Properties of the *Kingella kingae* PilC1 and PilC2 Proteins Have Differential Effects on Type IV Pilus-Mediated Adherence and Twitching Motility. J Bacteriol 195:886–895.

5. Yanisch-Perron C, Vieira J, Messing J. 1985. Improved M13 phage cloning vectors and host strains: nucleotide sequences of the M13mp18 and pUC19 vectors. Gene 33:103–119.

6. Hendrixson DR, Akerley BJ, DiRita VJ. 2001. Transposon mutagenesis of *Campylobacter jejuni* identifies a bipartite energy taxis system required for motility. Mol Microbiol 40:214–224.

7. Hamilton HL, Schwartz KJ, Dillard JP. 2001. Insertion-Duplication Mutagenesis of *Neisseria*: Use in Characterization of DNA Transfer Genes in the Gonococcal Genetic Island. J Bacteriol 183:4718–4726.

8. Porsch EA, Kehl-Fie TE, Geme JWSt. 2012. Modulation of *Kingella kingae* Adherence to Human Epithelial Cells by Type IV Pili, Capsule, and a Novel Trimeric Autotransporter. mBio 3.

9. Porsch EA, Allas MJ, Montoya NR, Muñoz VL, Tan L, Muszyński A, Azadi P, Hyland SN, Grimes CL, Kao T-T, Lowary TL, St Geme JW. 2025. Identification and characterization of

mono- and bifunctional galactan synthases in the pediatric pathogen *Kingella kingae*. J Biol Chem 301:110345.

10. Karimova G, Pidoux J, Ullmann A, Ladant D. 1998. A bacterial two-hybrid system based on a reconstituted signal transduction pathway. Proceedings of the National Academy of Sciences 95:5752–5756.
